# Pro-cognitive effect of 1MeTIQ on recognition memory in the ketamine model of schizophrenia in rats: the behavioural and neurochemical effects

**DOI:** 10.1007/s00213-020-05484-1

**Published:** 2020-02-20

**Authors:** Magdalena Białoń, Marcelina Żarnowska, Lucyna Antkiewicz-Michaluk, Agnieszka Wąsik

**Affiliations:** Department of Neurochemistry, Maj Institute of Pharmacology, Polish Academy of Sciences, Smętna 12, 31-343 Kraków, Poland

**Keywords:** Schizophrenia, Novel object recognition (NOR), 1,2,3,4-Tetrahydroisoquinoline (1MeTIQ), Ketamine, Olanzapine

## Abstract

**Rationale:**

Schizophrenia is a mental illness which is characterised by positive and negative symptoms and by cognitive impairments. While the major prevailing hypothesis is that altered dopaminergic and/or glutamatergic transmission contributes to this disease, there is evidence that the noradrenergic system also plays a role in its major symptoms.

**Objectives:**

In the present paper, we investigated the pro-cognitive effect of 1-methyl-1,2,3,4-tetrahydroisoquinoline (1MeTIQ) an endogenous neuroprotective compound, on ketamine-modelled schizophrenia in rats.

**Methods:**

We used an antagonist of NMDA receptors (ketamine) to model memory deficit symptoms in rats. Using the novel object recognition (NOR) test, we investigated the pro-cognitive effect of 1MeTIQ. Additionally, olanzapine, an atypical antipsychotic drug, was used as a standard to compare the pro-cognitive effects of the substances. In vivo microdialysis studies allowed us to verify the changes in the release of monoamines and their metabolites in the rat striatum.

**Results:**

Our study demonstrated that 1MeTIQ, similarly to olanzapine, exhibits a pro-cognitive effect in NOR test and enhances memory disturbed by ketamine treatment. Additionally, in vivo microdialysis studies have shown that ketamine powerfully increased noradrenaline release in the rat striatum, while 1MeTIQ and olanzapine completely antagonised this neurochemical effect.

**Conclusions:**

1MeTIQ, as a possible pro-cognitive drug, in contrast to olanzapine, expresses beneficial neuroprotective activity in the brain, increasing concentration of the extraneuronal dopamine metabolite, 3-methoxytyramine (3-MT), which plays an important physiological role in the brain as an inhibitory regulator of catecholaminergic activity. Moreover, we first demonstrated the essential role of noradrenaline release in memory disturbances observed in the ketamine-model of schizophrenia, and its possible participation in negative symptoms of the schizophrenia.

## Introduction

Schizophrenia is a devastating mental disorder that can result in cognitive deficits, including memory deficits, attentional deficits and executive functioning impairments, in addition to positive and negative symptoms (Heinrichs and Zakzanis 1998). These symptoms generally appear years before a clinical diagnosis (Lesh et al. 2011) and strongly influence patient quality of life; therefore, it is necessary to explore effective treatments for related cognitive impairments. Studies of memory in patients with schizophrenia have reported large deficits in verbal and visual features (Faraone et al. 2000) of recognition (Danion et al. 1999) and episodic memory (Krabbendam et al. 2001; Toulopoulou et al. 2003), both of which are part of declarative memory (Riedel and Blokland 2015). One of the most popular methods for investigating declarative memory processes in rodents is the novel object recognition (NOR) test—a non-rewarded, ethologically relevant paradigm that is based on the spontaneous exploratory behaviours of rodents and that does not require external motivation, punishment or training (Ennaceur and Delacour 1988; Ennaceur 2010; Cadinu et al. 2018). NOR test gained popularity as it is simple and cheap behavioural assay of memory that relies primarily on a rodent’s innate exploratory behaviour in the absence of externally applied rules, reinforcement or stressful training (Silvers et al. 2007; Reger et al. 2009; Mathiasen and DiCamillo 2010; Antunes and Biala 2012; Cadinu et al. 2018) . A preference for the novel object is evidence that the familiar object has been remembered by the animal. This form of memory is considered to be the rodent equivalent of human declarative (episodic) memory (Ennaceur 2010). In humans, the analogous task used to study declarative memory is a visual-paired comparison task (Sivakumaran et al. 2018), in which patients with schizophrenia show less accuracy in recognizing previously seen objects (Heckers et al. 2000). There are evidence that atypical antipsychotic drugs, such as olanzapine, improve cognitive functions, including memory (Wolff and Leander 2003; He et al. 2005; Mahmoud et al. 2019; Desamericq et al. 2014; Guo et al. 2011; Gurpegui et al. 2007; Meltzer and McGurk 1999; McGurk et al. 2004; Mutlu et al. 2011; Rajagopal et al. 2014; Babic et al. 2018) while other authors have shown that olanzapine can impair memory (Skarsfeldt 1996; Purdon et al. 2000; Levin et al. 2005).

Many theories of the molecular origins of schizophrenia symptoms have arisen, although the aetiology of the illness remains uncertain. Many networks and neurotransmitters may be involved in the pathophysiology of schizophrenia; however, no single neurotransmitter or system can explain the full picture of the heterogeneity of schizophrenia symptoms (Yang and Tsai 2017). In addition to the engagement of dopaminergic, serotonergic and noradrenergic systems, there is strong evidence from many research disciplines indicating the role of the hypofunction of N-methyl-D-aspartate receptors (NMDARs) as a primary neurophysiological contributor to schizophrenia (Weickert et al. 2013; Nakazawa et al. 2017; Bubeniková-Valesová et al. 2008; Duncan et al. 1999; Brandao-Teles et al. 2017; Balu 2016; Krystal et al. 1999). NMDAR antagonists such as ketamine, dizocilpine (MK-801) and phencyclidine (PCP), as well as transgenic animals, have become useful preclinical tools to model the illness (Gilmour et al. 2012; Neill et al. 2010; Coyle 2012; Bondi et al. 2012). 1-Methyl-1,2,3,4-tetrahydroisoquinoline (1MeTIQ) is an endogenous amine present and synthesised in the mammalian brain in low concentrations (Antkiewicz-Michaluk et al. 2014; Wąsik and Antkiewicz-Michaluk 2017). The 1MeTIQ present in the brain is a mixture of (R)- and (S)-enantiomers enzymatically synthesised from 2-phenylethylamine and pyruvate by 1MeTIQ-synthesizing enzyme, a membrane-bound protein localised in the mitochondrial synaptosomal fraction (Yamakawa et al. 1999). Previous studies have shown that 1MeTIQ has affinity for the agonistic form of dopamine receptors and acts as a reversible monoamine oxidase (MAOA and MAOB) inhibitor with neuroprotective, anti-depressive (Antkiewicz-Michaluk et al. 2001, 2006, 2007; Patsenka and Antkiewicz-Michaluk 2004; Wąsik et al. 2016) and anti-addictive (Wąsik et al. 2010) properties. Early studies on tetrahydroisoquinolines revealed their neuroleptic-like properties (Ginos and Doroski 1979), and our experiments confirmed that 1MeTIQ acts as a specific antagonist of agonistic conformations of dopamine receptors and may play an important physiological role as an inhibitory regulator that counteracts excessive stimulation of catecholaminergic systems (Antkiewicz-Michaluk et al. 2007; Vetulani et al. 2001, 2003). As we demonstrated earlier, 1MeTIQ antagonised hyperactivity evoked by MK-801 (Pietraszek et al. 2009) and by apomorphine (Antkiewicz-Michaluk et al. 2001). It is well known that the catabolism of dopamine to its final metabolite, homovanillic acid (HVA), occurs both intra- and extraneuronally. Dopamine present in neuronal cytoplasm is N-oxidised by the mitochondrial outer membrane enzyme MAO to form 3,4-dihydroxyphenylacetic acid (DOPAC), which is then extraneuronally *O*-methylated by catechol-*O*-methyltransferase (COMT) to form HVA. Dopamine released into the synaptic cleft may be taken up by DAT localised on dopamine terminals or may be extraneuronally *O*-methylated by COMT to form 3-methoxytyramine (3-MT), an extraneuronal metabolite. The formation of DOPAC is accompanied by the production of the most deleterious radicals: hydroxyl radicals (Chiueh et al. 1993). Therefore, the oxidative MAO-dependent pathway of dopamine catabolism may play an important role in the progressive and selective loss of dopaminergic neurons in the substantia nigra during the development of Parkinson’s disease. Alternatively, the enhanced catabolism of dopamine through COMT-dependent O-methylation leading to 3-MT accumulation may constitute an oxidative defence mechanism leading to neuroprotection (Miller et al. 1996; Antkiewicz-Michaluk et al. 2006). Furthermore, it was previously demonstrated that 3-MT, an extraneuronal dopamine metabolite, plays a physiological role in the brain as an inhibitory regulator of noradrenergic and dopaminergic activity (Antkiewicz-Michaluk et al. 2008).

In biochemical studies, it has been shown that the administration of 1MeTIQ leads to the activation of noradrenergic and serotonergic systems (Możdżeń et al. 2017), increases brain dopamine levels in animal models of depression and Parkinson’s disease and protects dopaminergic neurons from cell death (Antkiewicz-Michaluk et al. 2014). 1MeTIQ also acts as a low-affinity NMDA receptor antagonist (Kuszczyk et al. 2013). As a compound affecting multiple brain systems and networks, 1MeTIQ may be considered a potential agent useful in the treatment of the cognitive symptoms of schizophrenia.

Our recent study showed that 1MeTIQ exhibits anxiolytic properties in an animal model of schizophrenia (Wąsik et al. 2019); therefore, we decided to verify whether 1MeTIQ can improve cognitive functioning related to declarative memory.

The aim of the present study was to evaluate the pro-cognitive effect of acute 1MeTIQ administration in an animal model of schizophrenia (induced by an acute dose of ketamine) and to compare its action to that of olanzapine, an antipsychotic drug widely used in the treatment of schizophrenia (Aubry et al. 2000). Moreover, in an in vivo microdialysis study, changes in levels of neurotransmitters and their metabolites in the striatum (STR) were measured using high-performance liquid chromatography (HPLC) methods.

## Materials and methods

### Animals

All experiments were carried out in male Sprague-Dawley rats with an initial body weight of 250–275 g. The animals were kept in standard polyacrylic cages (5 animals/cage). All animals had free access to standard laboratory food and tap water and were kept at room temperature (22 °C) under an artificial light/dark cycle (12/12 h, lights on at 7:00 a.m.). One of two doses of 1MeTIQ (25 or 50 mg/kg, intraperitoneally (i.p.)) or olanzapine (3 mg/kg i.p.) was administered 30 min before the ketamine (20 mg/kg i.p.) injection. Control rats were treated with an appropriate vehicle (0.9% NaCl).

The NOR test was conducted 30 min after ketamine administration. All experimental groups consisted of 10 individuals. The doses of 1MeTIQ used in this experiment were based on our previous experience, while doses of olanzapine (Rogóż and Skuza 2011) and ketamine were based on the literature (Nikiforuk et al. 2016). Immediately after the behavioural experiments, the rats were killed by decapitation, and different structures of the brain were dissected out for later analysis. The experiments were carried out between 9.00 a.m. and 16.00 p.m. All experimental procedures were carried out in accordance with the Guide for the Care and Use of Laboratory Animals issued by the National Institutes of Health and received approval from the Bioethics Commission as being compliant with Polish law.

### Drugs

1-Methyl-1,2,3,4-tetrahydroisoquinoline (1MeTIQ) was synthesised by the Department of Drug Chemistry, Institute of Pharmacology Polish Academy of Sciences, Krakow, Poland. The purity of the compound was verified by the measurement of the melting point, and homogeneity was assessed on a chromatographic column. Ketamine (Biowet Puławy, Poland) was in the form of a solution for injection (ketamine hydrochloride). Olanzapine (Sigma-Aldrich, USA) was suspended in a 1% aqueous solution of Tween 80; 1MeTIQ was dissolved in sterile 0.9% NaCl solution and injected in a volume of 1 ml/kg.

### The NOR test

#### Apparatus

Animals were tested in a dimly lit (20 lux) open field made of black wood (60 × 60 × 25). After each animal performed the test, the box was cleaned with alcohol and allowed to dry.

#### Objects

Three objects of comparable size were used in the experiment. Object 1 (obj1) and object 2 (obj2), both used in the first trial, were visually the same (aluminium cans filled with sand and pasted with tape), and the third object (a glass bottle tightly closed and filled with water) was used as a novel object in the second trial. All objects were heavy enough to not be displaced by the animals.

#### Procedure

Animals were habituated to the box (without objects) for 5 min 24 h before the first trial of the test (Nikiforuk et al. 2013). The test consisted of two trials (T1 and T2, 5 min each) separated by 1-h intervals. In the first trial (T1), two identical objects were placed in the box 15 cm from the walls. After T1, rats were returned to their home cages. In T2, one of the objects was replaced with a novel object (NO). The location of the novel object was randomly determined for each animal to avoid the occurrence of place preference.

The exploration of an object was defined as looking at, licking, sniffing or touching the object but not leaning against or sitting or standing on the object (Nikiforuk et al. 2016).

The behaviour of the animals was recorded using a camera placed above the apparatus and connected to the Any-maze® tracking system.

#### Measured parameters

The exploration time of each object was manually assessed by the experimenter who was blinded to the experimental conditions. Based on the exploration time (E) of each object, two parameters were assessed: discrimination index (DI) = (E_NO_-E_FO_)/(E_NO_ + E_FO_); recognition index (RI) = E_NO_/(E_NO_ + E_FO_).

### Microdialysis study

Rats were anaesthetised with ketamine (75 mg/kg) and xylazine (10 mg/kg) and secured in a stereotaxic frame (Stoelting, USA). Vertical microdialysis guide cannulas (Intracerebral Guide Cannula with stylet; BAS Bioanalytical, USA) were implanted in the striatum (STR) according to the following stereotaxic coordinates: A/P + 1.0, L/M + 2.5 and V/D − 3.5 mm from bregma and the dura (G. Paxinos and C.H. Watson). Seven days after surgery, microdialysis probes (length 4 mm) were inserted into the cannulas, and the STR was perfused with artificial cerebrospinal fluid (aCSF), which consisted of 140 mM NaCl, 2.7 mM KCl, 1.2 mM CaCl_2_, 1 mM MgCl_2_, 0.3 mM NaH_2_PO_4_ and 1.7 mM Na_2_HPO_4_ (pH 7.4), at a flow rate of 1.5 μl/min maintained with a microinfusion pump (Stoelting, IL USA). Samples were collected from freely moving rats in 20-min intervals after a 3-h wash-out period. All dialysates were immediately frozen on dry ice (− 70 °C) until they were used in a biochemical assay.

Dopamine (DA), serotonin (5-HT), noradrenaline (NA) levels and the levels of their metabolites 3,4-dihydroxyphenylacetic acid (DOPAC), 3-methoxytyramine (3-MT), homovanillic acid (HVA) and normetanephrine (NM) in dialysates (20 μl) were analysed by HPLC with electrochemical detection. Chromatography was performed using an LC-10 AD pump (Shimadzu Europa GmbH, Warszawa, Poland), an LC-4B amperometric detector with a cross-flow detector cell (BAS, IN, USA), and a BDS-Hypersil C18 analytical column (3 × 100 mm^2^, a 3 μm, Thermo Electron Corp., UK). The mobile phase was composed of 0.1 M monochloracetic acid adjusted to pH = 3.7 with 3 M sodium hydroxide, 0.5 mM EDTA, 25 mg/l 1-octanesulfonic acid sodium salt, 5.7% methanol and 2.5% acetonitrile. The flow rate was 0.5 ml/min, and the applied potential of a 3 mm glassy carbon electrode was + 600 mV with a sensitivity of 2 nA/V. The chromatographic data were processed by Chromax 2001 (Pol-Lab, Warszawa, Poland) software run on a personal computer. The values were not corrected for an in vitro probe recovery, which was approximately 15%.

### Statistical analysis

The exploration times in the behavioural test were analysed with Student’s *t* test. DI and PI factors were compared with one-way analysis of variance (ANOVA) followed, when appropriate, by Duncan’s post hoc test. The data from the microdialysis study were analysed with one-way ANOVA for repeated measures followed, when appropriate, by Duncan’s post hoc tests. The results were considered statistically significant when *p* < 0.05.

## Results

### NOR test

#### The effect of 1MeTIQ (25 mg/kg i.p.) on ketamine-induced cognitive impairment in the novel object recognition task

**Fig. 1:**
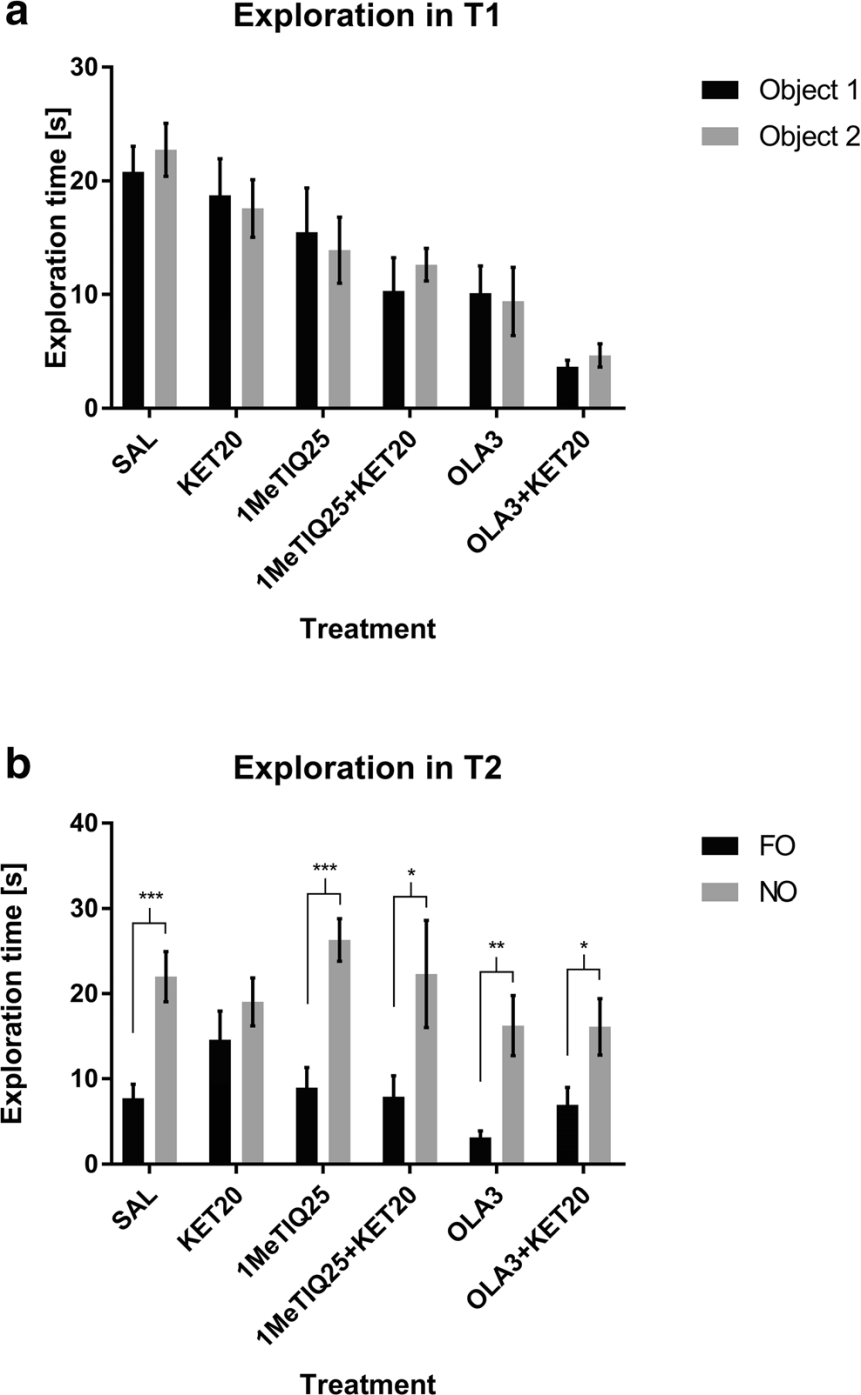
The effects of 1MeTIQ (25 mg/kg i.p.), olanzapine (3 mg/kg i.p.) and ketamine on exploration times for object 1 and object 2 in the acquisition trial (T1) (**a**) and in the retention trial (T2) (**b**) in the novel object recognition task. The data are shown as the mean ± SEM. *N* = 9–10 rats per group. Statistical significance: *p < 0.05, **p < 0.01, ***p < 0.001 significant differences in exploration times for the novel object (NO) and familiar object (FO)

Student’s *t* test showed no significant differences between the exploration times for object 1 and object 2 in all groups in T1 of the NOR test (Fig. 1a).

The same test showed significant differences (*p* < 0.001) in the exploration times for the NO and FO in the control (saline) group in the T2 phase. In ketamine-treated animals, no significant differences were observed in the exploration times for the objects. We observed statistically significant differences in the exploration times for the NO and FO in groups treated with 1MeTIQ (*p* < 0.001), combined 1MeTIQ and ketamine (*p* < 0.05), olanzapine (*p* < 0.01) and combined olanzapine and ketamine (*p* < 0.05) (Fig. 1b).

**Fig. 2:**
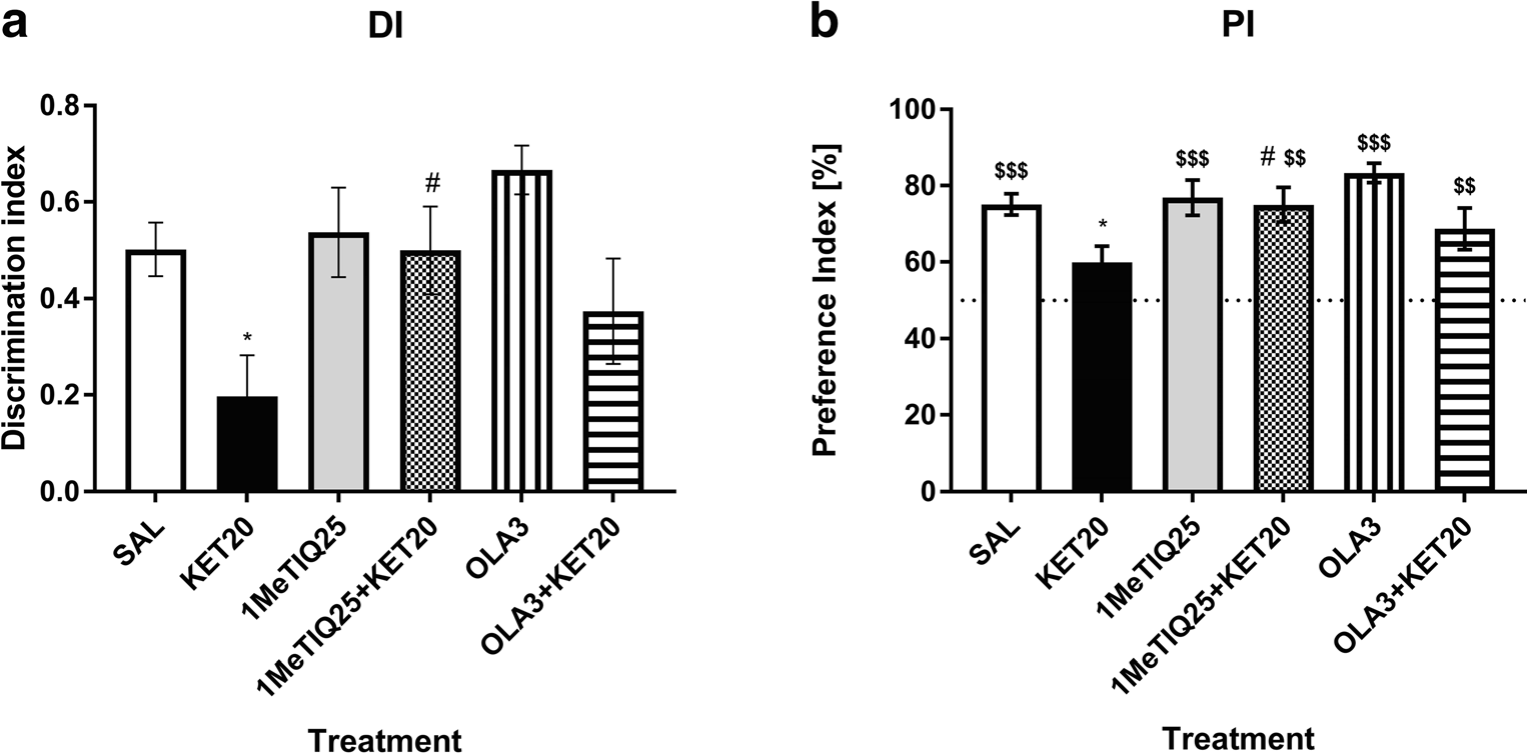
The effect of 1MeTIQ (25 mg/kg i.p.) and olanzapine (3 mg/kg i.p.) on ketamine-induced cognitive impairment in the novel object recognition task. The data are shown as the mean ± SEM of the discrimination index (DI) (**a**) and preference index (PI) (**b**) in the retention trial (T2), conducted 1 h following the acquisition trial (T1). *N* = 9–10 rats per group. Dotted line (**b**) indicates a chance level (50%). Statistical significance: *p < 0.05, **p < 0.01 significant reduction in the DI and PI compared with those in the vehicle-treated group; ^#^*p* < 0.05, ^##^*p* < 0.01 significant improvement in the DI or PI compared with that in the ketamine-treated group. $*p* < 0.05, $$*p* < 0.01, $$$*p* < 0.001 significant difference between PI and the chance level

The statistical analysis of the behavioural results showed that the discrimination index (DI) significantly differed among groups (*F*[5,44] = 3.55, *p* < 0.01). The post hoc test showed a significant effect (*p* < 0.05) of ketamine on the DI value. 1MeTIQ given with ketamine produced significant effects (*p* < 0.05) and completely blocked ketamine-induced changes. However, olanzapine given with ketamine did not produce significant changes in the DI (Fig. 2a).

The same analysis showed a significant effect (*F*[5,44] = 3.55, *p* < 0.01) of treatment on the preference index (PI). Ketamine significantly (*p* < 0.05) lowered the PI value compared to that in the control group. 1MeTIQ given with ketamine significantly (*p* < 0.05) changed the PI value and restored it to the control level. Olanzapine administered with ketamine did not significantly affect the PI (Fig. 2b).

Student’s *t* test showed significant (*p* < 0.001) difference in PI between saline group and the chance level (50%). PI in ketamine-treated group did not significantly differ from a chance. Both, 1MeTIQ (25 mg/kg) and olanzapine given alone significantly (*p* < 0.001) changed PI compared to a chance. Value of PI in both combined groups, significantly (*p* < 0.01) differed from the chance level (Fig. 2b).

#### The effect of 1MeTIQ (50 mg/kg i.p.) on ketamine-induced cognitive impairment in the novel object recognition task

**Fig. 3:**
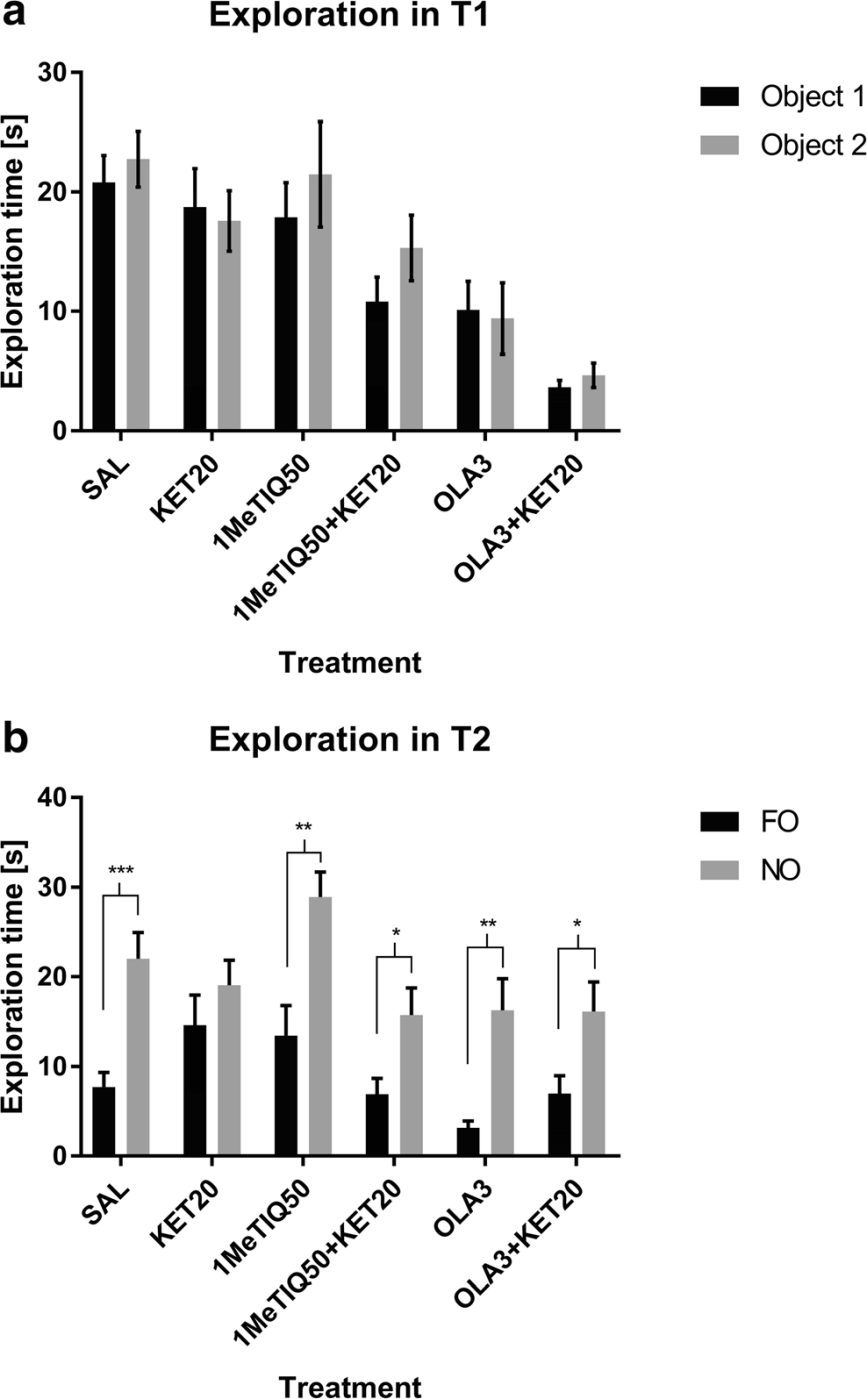
The effects of 1MeTIQ (50 mg/kg i.p.), olanzapine (3 mg/kg i.p.) and ketamine on exploration times for object 1 and object 2 in the acquisition trial (T1) (**a**) and in the retention trial (T2) (**b**) in the novel object recognition task. The data are shown as the mean ± SEM. *N* = 9–10 rats per group. Statistical significance: *p < 0.05, **p < 0.01, ***p < 0.001 significant differences in exploration time for novel object (NO) and familiar object (FO)

Student’s *t* test showed no significant changes in the exploration times for object 1 and object 2 in all groups in the T1 phase of the NOR test (Fig. 3a).

The same statistical analysis showed significant changes in exploration times for the FO and NO in the T2 phase of the NOR test in the saline (control) group (*p* < 0.001). In ketamine-treated animals, no significant difference was observed. Statistically significant changes in exploration times for the FO and NO occurred in groups treated with 1MeTIQ (*p* < 0.01), combined 1MeTIQ and ketamine (*p* < 0.05), olanzapine (*p* < 0.01) and combined olanzapine and ketamine (*p* < 0.05) (Fig. 3b).

**Fig. 4:**
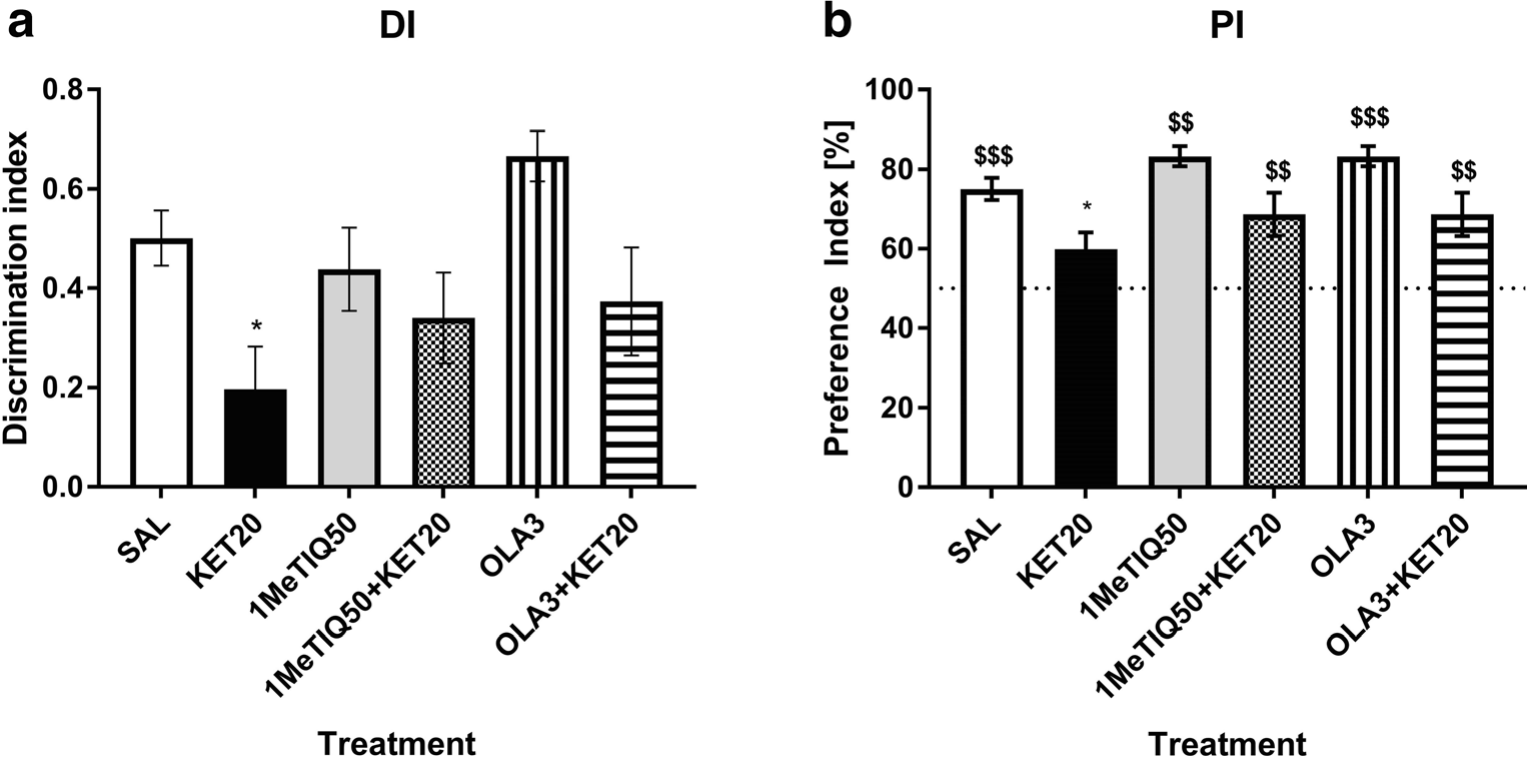
The effect of 1MeTIQ (50 mg/kg i.p.) and olanzapine (3 mg/kg i.p.) on ketamine-induced cognitive impairment in the novel object recognition task. The data are shown as the mean ± SEM of the discrimination index (DI) (**a**) and preference index (PI) (**b**) in the retention trial (T2), conducted 1 h following the acquisition trial (T1). *N* = 9–10 rats per group. Dotted line (**b**) indicates a chance level (50%). Statistical significance: *p < 0.05, **p < 0.01 significant reduction in the DI and PI compared with those in the vehicle-treated group; ^#^*p* < 0.05, ^##^*p* < 0.01 significant improvement in the DI or PI compared with that in the ketamine-treated group. $*p* < 0.05, $$*p* < 0.01, $$$*p* < 0.001 significant difference between PI and the chance level

Statistical analysis showed a significant effect (*F*[5,45] = 3.44, *p* < 0.05) of treatment on the DI. Post hoc analysis showed that ketamine significantly (*p* < 0.05) lowered the DI value. However, neither 1MeTIQ (50 mg/kg) nor olanzapine, when given with ketamine, could reverse this effect (Fig. 4a).

The same analysis showed a significant effect (*F*[5,45] = 3.44, *p* < 0.01) of treatment on the PI value. Ketamine significantly (*p* < 0.05) affected the PI; however, 1MeTIQ and olanzapine did not reverse its effect in the combined groups (Fig. 4b).

Student’s t test showed significant (*p* < 0.001) difference in PI between the control group and the chance level (50%). PI in ketamine-treated group did not significantly differ from a chance. Both, 1MeTIQ (50 mg/kg) and olanzapine given alone significantly (*p* < 0.01 and *p* < 0.001, respectively) changed PI when compared to a chance. Value of PI in both combined groups, significantly (*p* < 0.01) differed from the chance level (Fig. 4b).

### In vivo microdialysis study

#### The effect of the acute administration of 1MeTIQ (25 mg/kg i.p.) on ketamine-induced changes in dopamine release and levels of its metabolites in the rat striatum

**Fig. 5:**
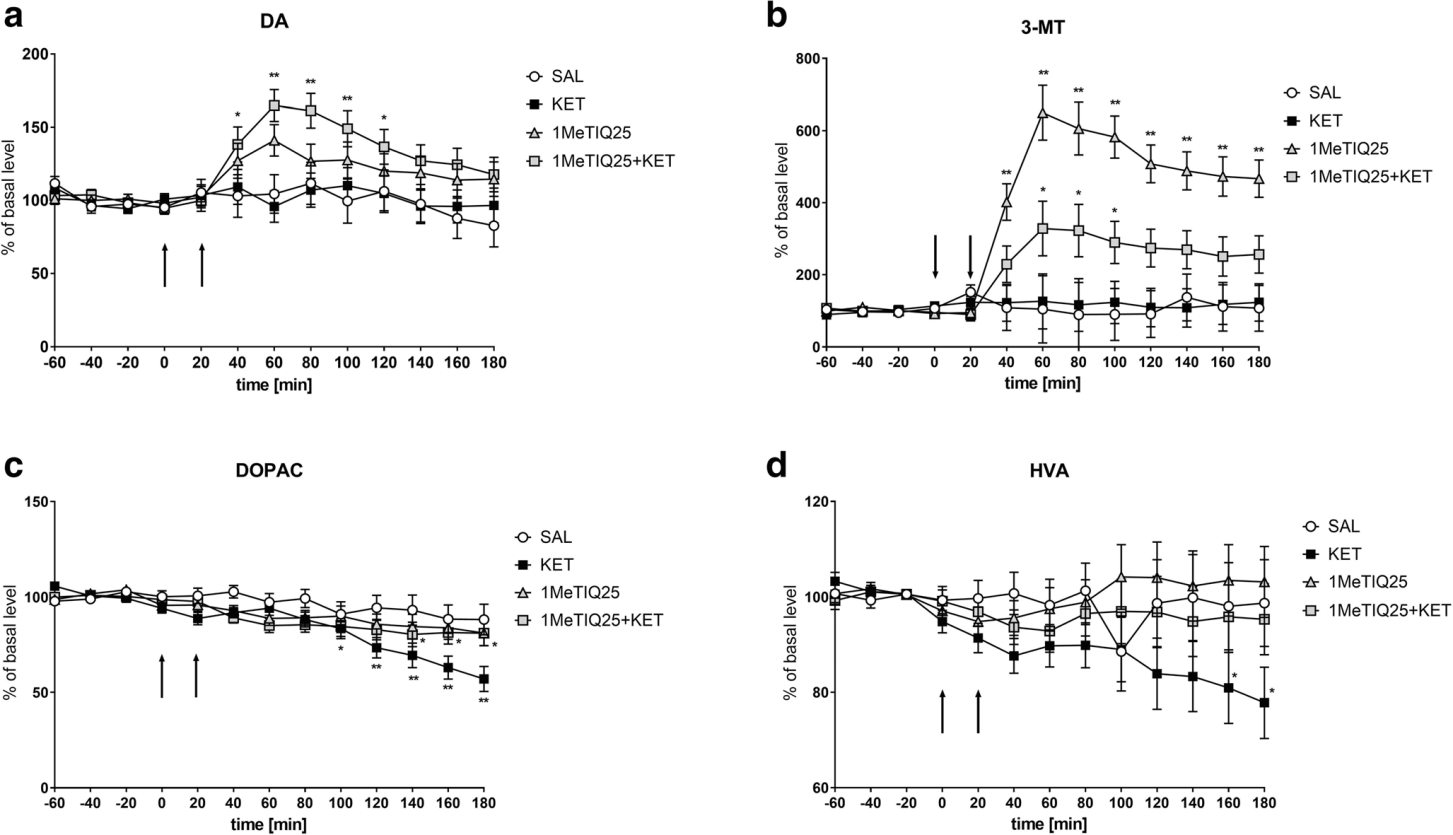
The effect of the acute administration of 1MeTIQ (25 mg/kg i.p.) on ketamine-induced changes in dopamine release and levels of its metabolites in the rat striatum. Control samples were collected from “– 60” to “0;” then, 1MeTIQ (25 mg/kg; at timepoint “0”) or ketamine (20 mg/kg; at timepoint “20”) was administered i.p. Dialysates were collected every 20 min. In the combined treatment group, 1MeTIQ was injected 20 min before ketamine administration. The concentrations of dopamine (DA) (**a**) and its metabolites 3-MT (**b**), DOPAC (**c**) and HVA (**d**) were measured. The basal level of dopamine in the striatum was 8.1 ± 0.8 pg/20 μl. The data are expressed as the means ± SEM (*n* = 5–6). Statistical significance: *p < 0.05, **p < 0.01 from the basal value (Duncan’s test).

The mean control basal extracellular concentration of dopamine in dialysates obtained from the striatum was approximately 8.1 ± 0.8 (pg/20 μl). One-way ANOVA for repeated measures indicated a significant effect of treatment on DA release (*F*[3,18] = 3.18; *p* < 0.05). Duncan’s test demonstrated that a single dose of ketamine did not change the dopamine concentration in the rat striatum (Fig. 5a). Additionally, an acute dose of 1MeTIQ (25 mg/kg i.p.) did not cause significant changes in dopamine release. In contrast, combined 1MeTIQ (25 mg/kg i.p.) and ketamine treatment produced a significant elevation in the dopamine concentration (approx. 160%; *p* < 0.01) (Fig. 5a).

Statistical analysis showed a significant effect of treatment on the 3-MT concentration (*F*[3,18] = 10.20; *p* < 0.01). Duncan’s test indicated that acute ketamine administration did not change 3-MT levels (Fig. 5b). In contrast, 1MeTIQ (25 mg/kg i.p.) administration increased the 3-MT concentration approximately 350%, while in the combined treatment group (1MeTIQ + ketamine), 3-MT levels were elevated more than 650% (Fig. 5b).

One-way ANOVA for repeated measures indicated no significant effect of treatment on the DOPAC concentration (*F*[3,18] = 1.96; N.S.), while the effect of TIME (*F*[12,216] = 24.28; *p* < 0.01) and the interaction between TIME and treatment (*F*[36,216] = 2.7; *p* < 0.01) were significant. Post hoc analysis indicated that a single dose of ketamine significantly decreased the level of DOPAC (up to 40%) (Fig. 5c). 1MeTIQ (25 mg/kg i.p.) administered alone and combined with ketamine produced a weaker effect, with the DOPAC concentration being reduced by approximately 15% (Fig. 5c).

Statistical analysis showed no significant effect of treatment on the HVA concentration (*F*[3,18] = 1.3; N.S.) while the effect of TIME (*F*[12,216] = 1.9; *p* < 0.05) and the interaction between TIME and treatment (*F*[36,216] = 1.53; p < 0.05) were significant. Duncan’s test demonstrated that treatment with ketamine significantly reduced HVA levels by approximately 20% (Fig. 5d). The same analysis indicated that 1MeTIQ given alone and in combination with ketamine did not change the HVA concentration in the rat striatum (Fig. 5d).

#### The effect of the acute administration of 1MeTIQ (25 mg/kg i.p.) on ketamine-induced changes in noradrenaline release and levels of its metabolite in the rat striatum

**Fig. 6:**
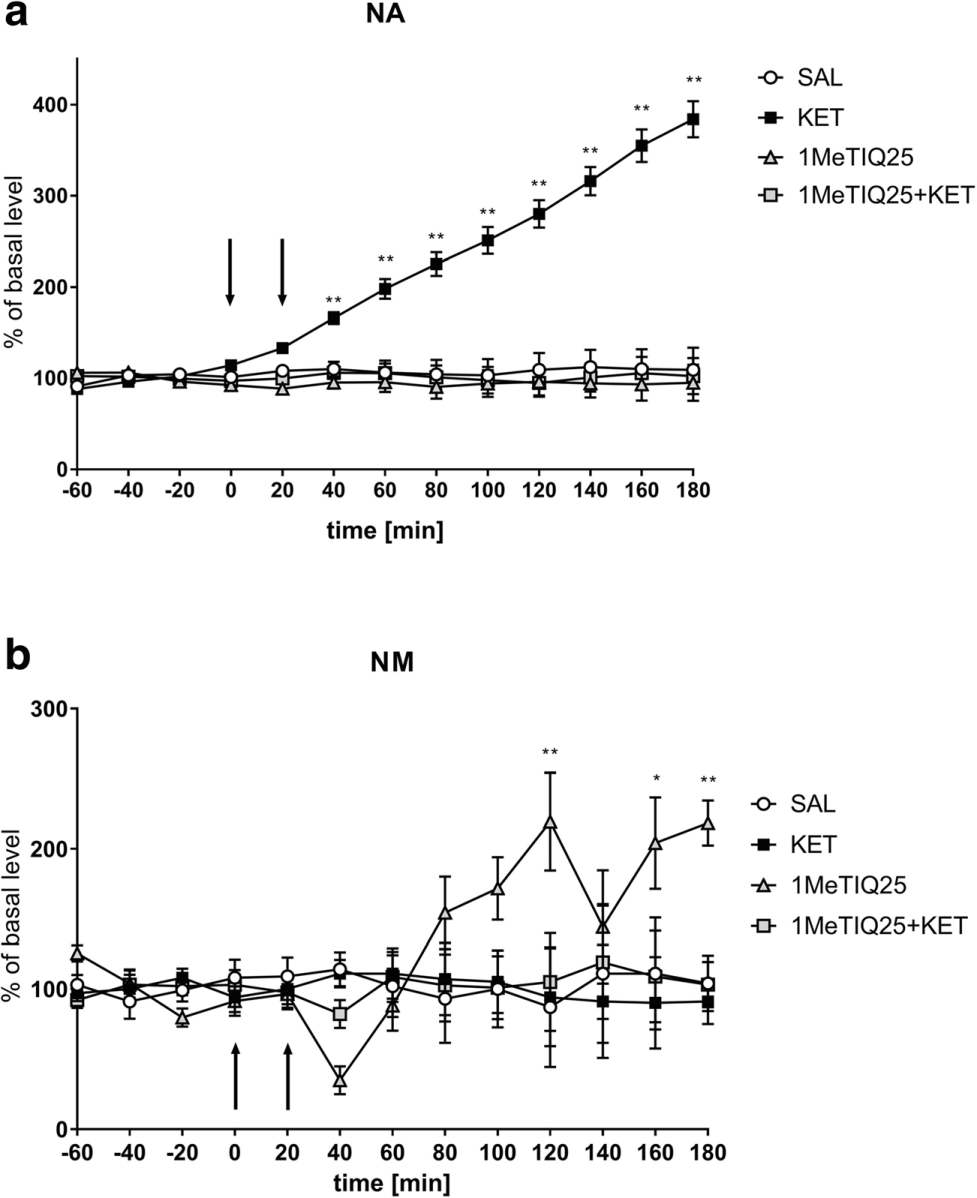
The effect of the acute administration of 1MeTIQ (25 mg/kg i.p.) on ketamine-induced changes in noradrenaline release and levels of its metabolite in the rat striatum. Control samples were collected from “– 60” to “0;” then, 1MeTIQ (25 mg/kg; at timepoint “0”) or ketamine (20 mg/kg; at timepoint “20”) was administered i.p. Dialysates were collected every 20 min. In the combined treatment group, 1MeTIQ was injected 20 min before ketamine administration. The concentration of noradrenaline (NA) (**a**) and its metabolite NM (**b**) were measured. The data are expressed as the means ± SEM (*n* = 5–6). Statistical significance: *p < 0.05, **p < 0.01 from the basal value (Duncan’s test)

Statistical analysis showed a significant effect of treatment on NA release (*F*[3,18] = 38.95; *p* < 0.01). Duncan’s test demonstrated a very large, time-dependent increase in NA release after an acute dose of ketamine (up to 400%), and this effect was completely antagonised by a single dose of 1MeTIQ (25 mg/kg i.p.); in the combined group, the level of NA was similar to that in the control (saline) group (Fig. 6a).

The same analysis showed no significant effect of treatment on the NM concentration (*F*[3,18] = 1.44; N.S.), while both the effect of TIME (*F*[12,216] = 2.04; *p* < 0.05) and the interaction between TIME and treatment (*F*[36,216] = 2.71; *p* < 0.01) were significant. Duncan’s test demonstrated that ketamine administration (alone or in combination with 1MeTIQ) did not change NM levels (Fig. 6b). In contrast, 1MeTIQ (25 mg/kg i.p.) administration showed an increase in the NM concentration of approximately 200% (Fig. 6b).

#### The effect of the acute administration of 1MeTIQ (50 mg/kg i.p.) on ketamine-induced changes in dopamine release and levels of its metabolites in the rat striatum

**Fig. 7:**
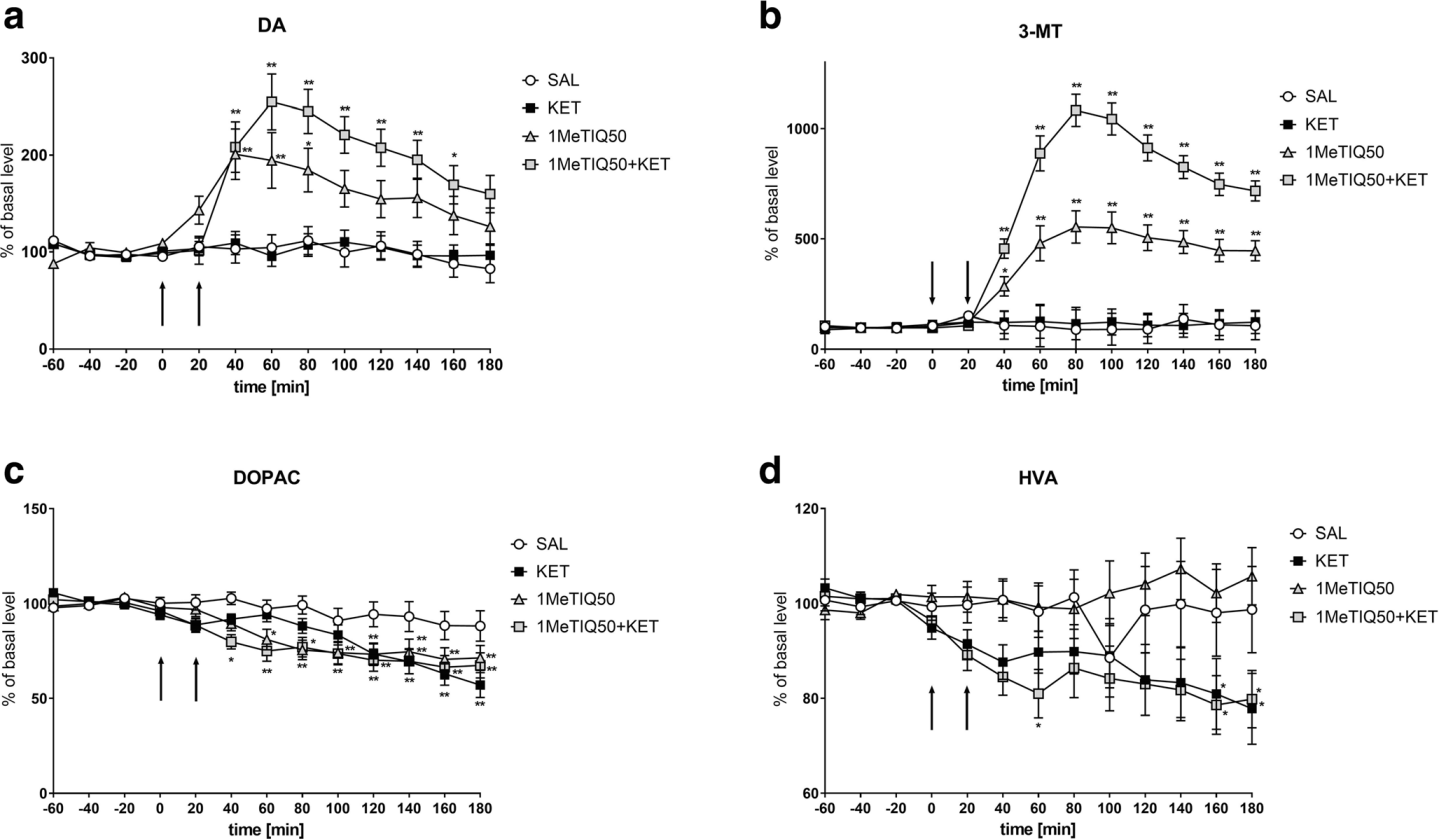
The effect of the acute administration of 1MeTIQ (50 mg/kg i.p.) on ketamine-induced changes in dopamine release and levels of its metabolites in the rat striatum. Control samples were collected from “– 60” to “0;” then, 1MeTIQ (50 mg/kg; at timepoint “0”) or ketamine (20 mg/kg; at timepoint “20”) was administered i.p. Dialysates were collected every 20 min. In the combined treatment group, 1MeTIQ was injected 20 min before ketamine administration. The concentration of dopamine (DA) (**a**) and its metabolites 3-MT (**b**), DOPAC (**c**) and HVA (**d**) were measured. The basal level of dopamine in the striatum was 8.1 ± 0.8 pg/20 μl. The data are expressed as the means ± SEM (*n* = 5–6). Statistical significance: *p < 0.05, **p < 0.01 from the basal value (Duncan’s test)

Statistical analysis showed a significant effect of treatment on DA release (*F*[3,18] = 5.45; *p* < 0.01). A post hoc test demonstrated that a single dose of ketamine did not change DA release in the rat striatum (Fig. 7a). The same analysis showed that an acute dose of 1MeTIQ (50 mg/kg i.p.) significantly increased DA release (up to 200%) (Fig. 7a). At the same time, combined 1MeTIQ (50 mg/kg i.p.) and ketamine treatment produced a stronger increase in the DA concentration in the extracellular space (up to 250%) (Fig. 7a).

One-way ANOVA for repeated measures indicated a significant effect of treatment on the 3-MT concentration (*F*[3,18] = 35.33; *p* < 0.01). Duncan’s test showed that acute ketamine administration did not change the level of 3-MT (Fig. 7b). In contrast, the administration of 1MeTIQ (50 mg/kg i.p.) increased the 3-MT concentration approximately 500%, while in the combined treatment group (1MeTIQ + ketamine), the 3-MT level was elevated above 1000% (Fig. 7b).

One-way ANOVA for repeated measures indicated no significant effect of treatment on DOPAC concentration (*F*[3,18] = 2.57; N.S.), while both the effect of TIME (*F*[12,216] = 35.38; *p* < 0.01) and the interaction between TIME and treatment (*F*[36,216] = 2.73; *p* < 0.01) were significant. Post hoc analysis indicated that either a single dose of ketamine or 1MeTIQ (50 mg/kg i.p.) significantly decreased the level of DOPAC (up to 40%) (Fig. 7c). A similar effect was observed in the combined group (1MeTIQ + ketamine) (Fig. 7c).

The same analysis demonstrated no significant effect of treatment on HVA concentration (*F*[3, 18] = 2.93; N.S.), while both the effect of TIME (*F*[12,216] = 5.05; *p* < 0.01) and the interaction between TIME and treatment (*F*[36,216] = 2.6; *p* < 0.01) were significant. Duncan’s test demonstrated that treatment with ketamine significantly reduced the level of HVA by approximately 20% (Fig. 7d). A similar effect was observed after combined treatment with 1MeTIQ (50 mg/kg i.p.) and ketamine (Fig. 7d).

#### The effect of the acute administration of 1MeTIQ (50 mg/kg i.p.) on ketamine-induced changes in noradrenaline release and levels of its metabolite in the rat striatum

**Fig. 8:**
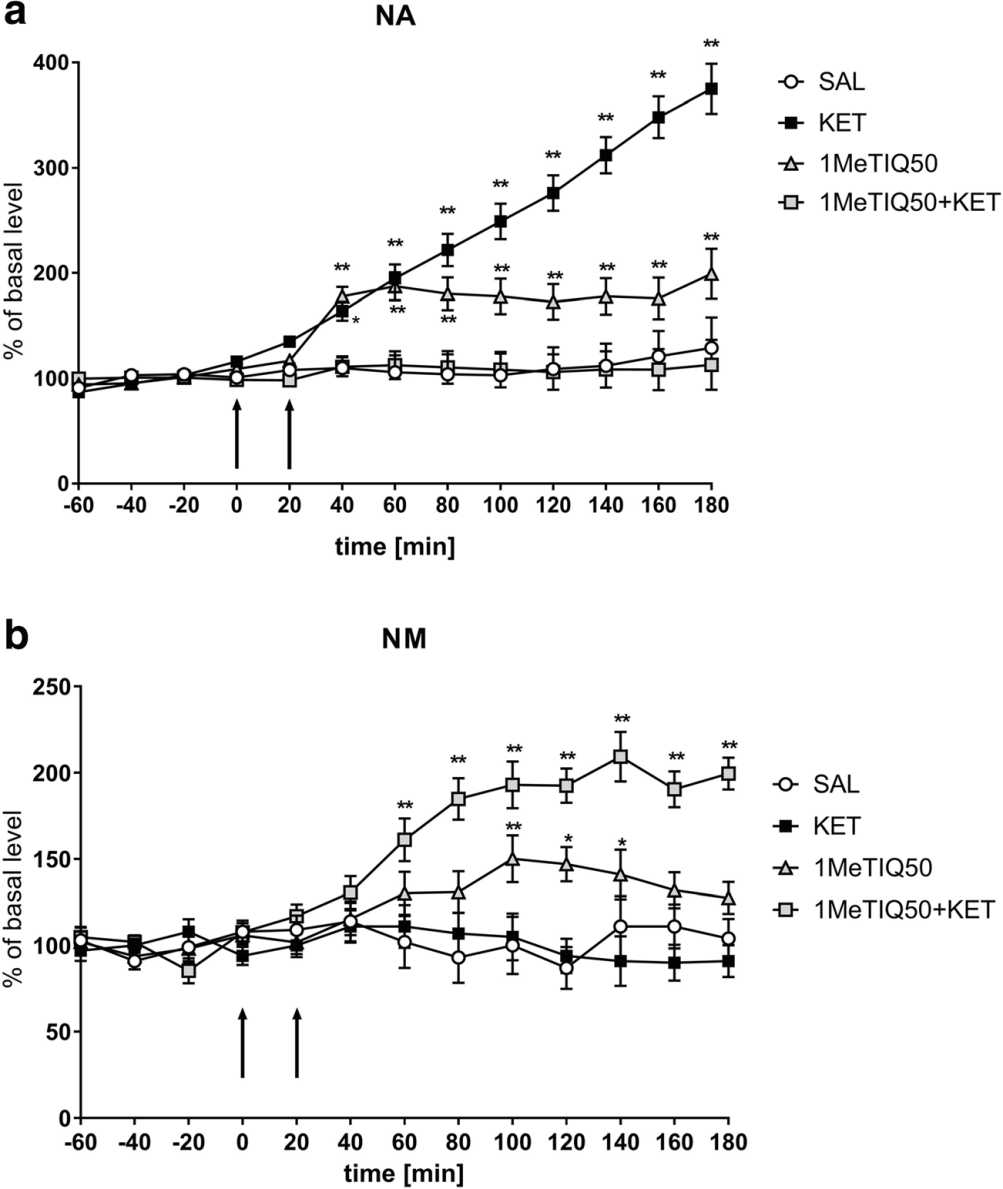
The effect of the acute administration of 1MeTIQ (50 mg/kg i.p.) on ketamine-induced changes in noradrenaline release and levels of its metabolite in the rat striatum. Control samples were collected from “– 60” to “0;” then, 1MeTIQ (50 mg/kg; at timepoint “0”) or ketamine (20 mg/kg; at timepoint “20”) was administered i.p. Dialysates were collected every 20 min. In the combined treatment group, 1MeTIQ was injected 20 min before ketamine administration. The concentration of noradrenaline (NA) (**a**) and its metabolite NM (**b**) were measured. The data are expressed as the means ± SEM (*n* = 5–6). Statistical significance: *p < 0.05, **p < 0.01 from the basal value (Duncan’s test)

One-way ANOVA for repeated measures indicated a significant effect of treatment on NA release (*F*[3,18] = 21.44; *p* < 0.01). Duncan’s test demonstrated a strong, time-dependent increase in NA release after an acute dose of ketamine (up to 390%) (Fig. 8a), and this effect was completely antagonised by a single dose of 1MeTIQ (50 mg/kg i.p.). In the combined group, the level of NA was similar to that in the control (saline) group (Fig. 8a). In addition, 1MeTIQ (50 mg/kg i.p.) given alone produced an increase in NA release of approximately 200% (Fig. 8a).

The same analysis showed a significant effect of treatment on NM concentration (*F*[3,18] = 12.77; *p* < 0.01). A post hoc test demonstrated that ketamine administration did not change NM levels (Fig. 8b) In contrast, both 1MeTIQ (50 mg/kg i.p.) given alone (approx. 150%) and combined with ketamine (approx. 200%) induced a significant increase in NM levels in the extracellular space (Fig. 8b).

#### The effect of the acute administration of olanzapine (3 mg/kg i.p.) on ketamine-induced changes in dopamine release and levels of its metabolites in the rat striatum

**Fig. 9:**
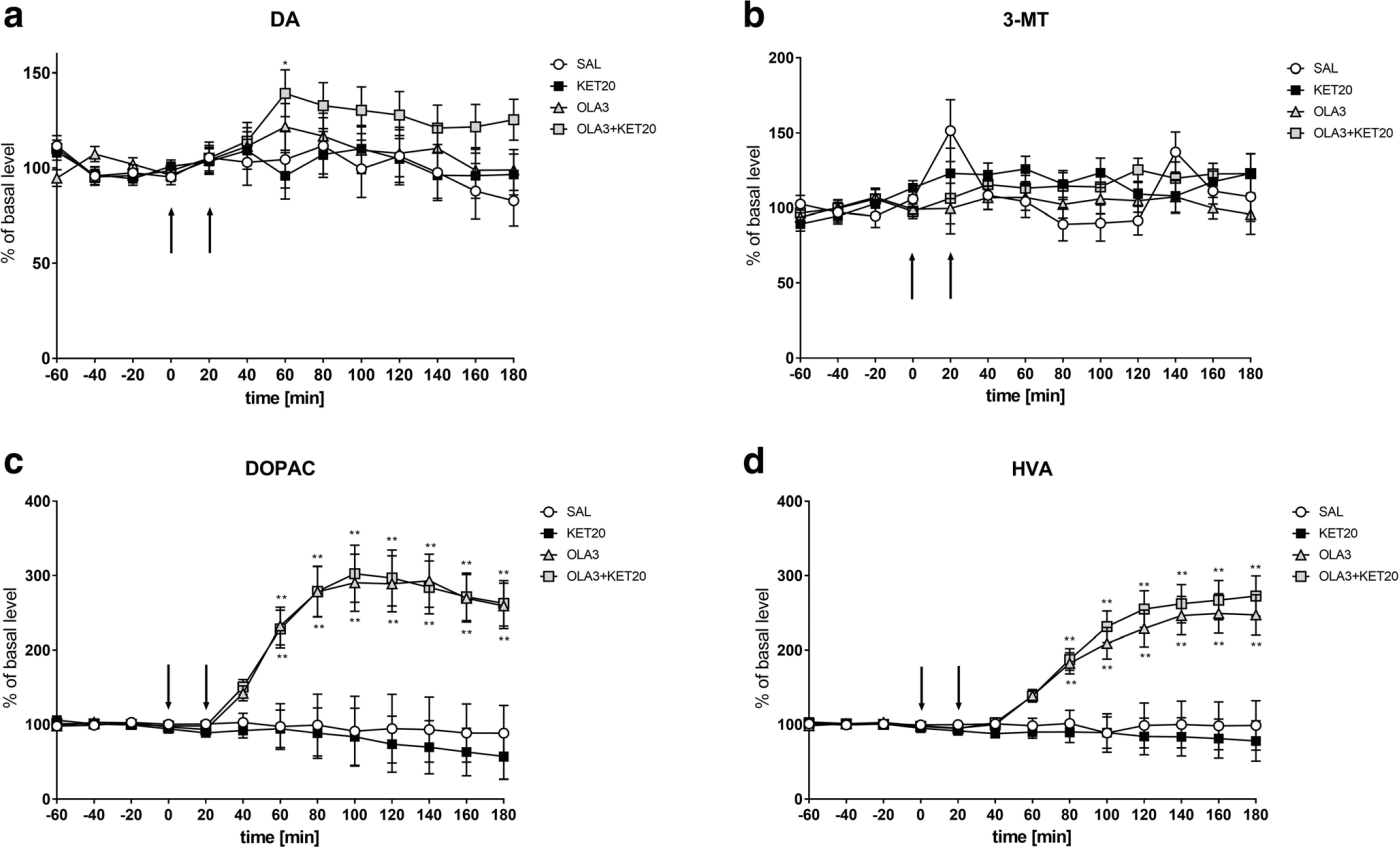
The effect of the acute administration of olanzapine (3 mg/kg i.p.) on ketamine-induced changes in dopamine release and levels of its metabolites in the rat striatum. Control samples were collected from “– 60” to “0;” then, olanzapine (3 mg/kg; at timepoint “0”) or ketamine (20 mg/kg; at timepoint “20”) was administered i.p. Dialysates were collected every 20 min. In the combined treatment group, olanzapine was injected 20 min before ketamine administration. The concentration of dopamine (DA) (**a**) and its metabolites 3-MT (**b**), DOPAC (**c**) and HVA (**d**) were measured. The basal level of dopamine in the striatum was 8.1 ± 0.8 pg/20 μl. The data are expressed as the means ± SEM (*n* = 5–6). Statistical significance: *p < 0.05, **p < 0.01 from the basal value (Duncan’s test)

One-way ANOVA for repeated measures showed no significant effect of treatment on DA release (*F*[3,18] = 1.27; N.S.), while the effect of TIME (*F*[12,216] = 2.91; *p* < 0.01) was significant. However, the interaction between TIME and treatment (*F*[36,216] = 1.16; N.S.) was not significant. A post hoc test demonstrated that a single dose of ketamine or olanzapine did not change DA release in the rat striatum (Fig. 9a). A similar effect was observed after combined treatment with ketamine and olanzapine (Fig. 9a).

Statistical analysis indicated no significant effect of treatment on the 3-MT concentration (*F*[3,18] = 1.25; N.S.), while the effect of TIME (*F*[12,216] = 2.63; *p* < 0.01) was significant. However, the interaction between TIME and treatment (*F*[36,216] = 1.4; N.S.) was not significant. Duncan’s test showed that acute ketamine or olanzapine administration did not change 3-MT levels (Fig. 9b). Additionally, combined treatment with ketamine and olanzapine did not change 3-MT levels (Fig. 9b).

One-way ANOVA for repeated measures indicated a significant effect of treatment on the DOPAC concentration (*F*[3,18] = 10.27; *p* < 0.01). Additionally, the effect of TIME (*F*[12,216] = 19.54; *p* < 0.01) and the interaction between TIME and treatment (*F*[36,216] = 9.56; *p* < 0.01) were significant. Post hoc analysis indicated that both a single dose of olanzapine and combined ketamine and olanzapine treatment significantly increased DOPAC levels (up to 300%) (Fig. 9c), while ketamine alone did not change this parameter.

The same analysis demonstrated a significant effect of treatment on the HVA concentration (*F*[3,18] = 12.23; *p* < 0.01), the effect of TIME (*F*[12,216] = 27.6; *p* < 0.01) and the interaction between TIME and treatment (*F*[36,216] = 12.04; *p* < 0.01). Duncan’s test demonstrated that both a single dose of olanzapine and combined ketamine and olanzapine treatment significantly increased HVA levels (approx. 250%) (Fig. 9d), while ketamine alone did not change this parameter (Fig. 9d).

#### The effect of the acute administration of olanzapine (3 mg/kg i.p.) on ketamine-induced changes in noradrenaline release and levels of its metabolite in the rat striatum

**Fig. 10:**
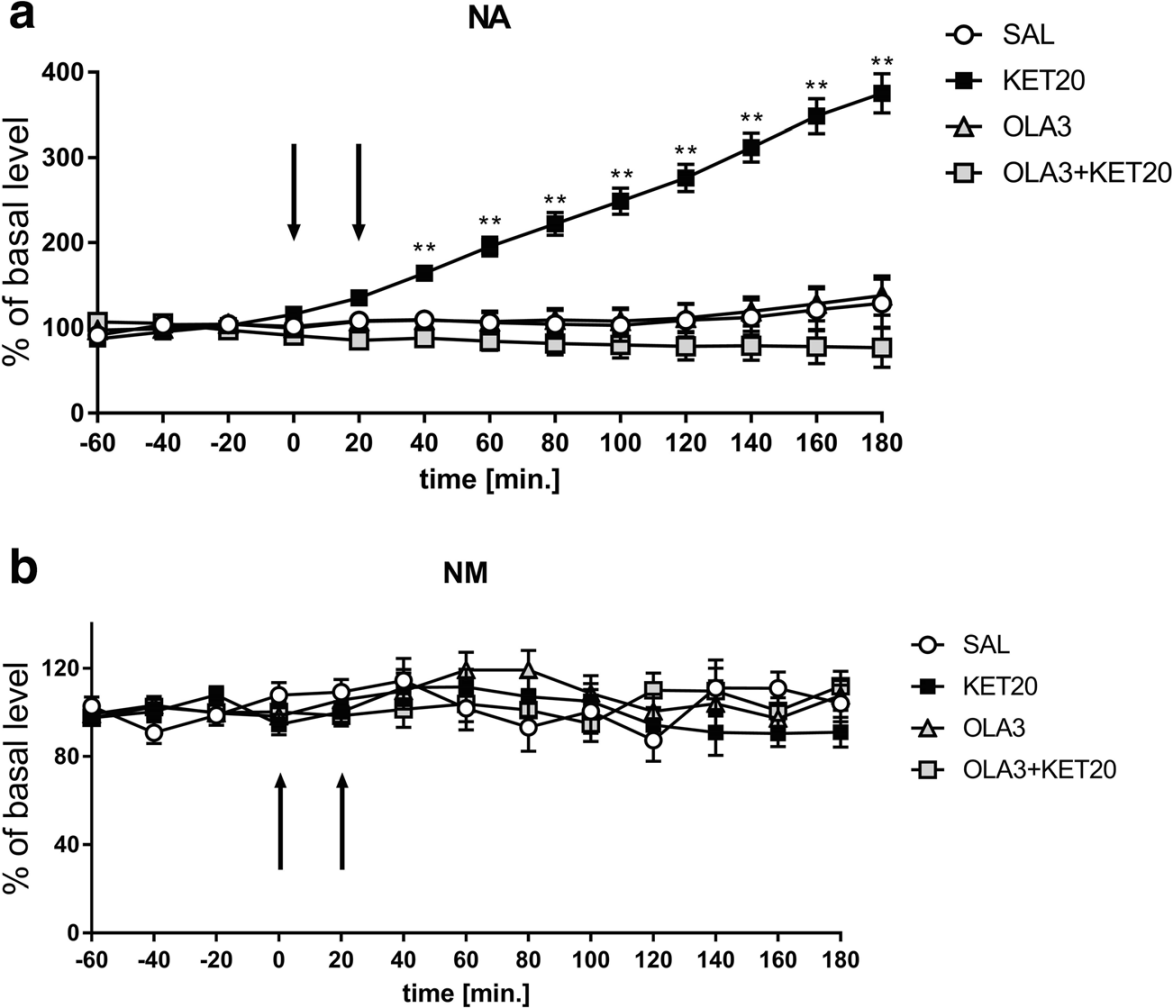
The effect of the acute administration of olanzapine (3 mg/kg i.p.) on ketamine-induced changes in noradrenaline release and levels of its metabolite in the rat striatum. Control samples were collected from “– 60” to “0;” then, olanzapine (3 mg/kg; at timepoint “0”) or ketamine (20 mg/kg; at timepoint “20”) was administered i.p. Dialysates were collected every 20 min. In the combined treatment group, olanzapine was injected 20 min before ketamine administration. The concentration of noradrenaline (NA) (**a**) and its metabolite NM (**b**) were measured. The data are expressed as the means ± SEM (*n* = 5–6). Statistical significance: *p < 0.05, **p < 0.01 from the basal value (Duncan’s test)

One-way ANOVA for repeated measures indicated a significant effect of treatment on NA release (*F*[3,18] = 33.31; *p* < 0.01). Additionally, the effect of TIME (*F*[12,216] = 30.81; *p* < 0.01) and the interaction between TIME and treatment (*F*[36,216] = 27.32; *p* < 0.01) were significant. Duncan’s test demonstrated a strong, time-dependent increase in NA release after an acute dose of ketamine (up to 380%) (Fig. 10a). This effect was completely antagonised by a single dose of olanzapine. In the combined group, NA levels were approximately 30% below the control level (Fig. 10a). At the same time, olanzapine alone did not change NA release (Fig. 10a).

The same analysis showed no significant effect of treatment on the NM concentration (*F*[3,18] = 0.31; N.S.) in the rat striatum. Similarly, the effect of TIME (*F*[12,216] = 1.34; N.S.) and the interaction between TIME and treatment (*F*[36,216] = 1.34; N.S.) were not significant (Fig. 10b) Table 1.

**Table 1:** Table summarizing the results obtained in in vivo microdialysis studies

	DA	DOPAC	3-MT	HVA	NA	NM
Saline	–	–	–	–	–	–
KET	–	↓	–	↓	↑↑	–
1MeTIQ25	–	↓	↑↑	–	–	↑
1MeTIQ50	↑	↓	↑↑	–	↑	↑
OLA	–	↑	–	↑	–	–
1MeTIQ25 + KET	↑	↓	↑	–	Antagonism to ketamine	–
1MeTIQ50 + KET	↑	↓	↑↑	↓	Antagonism to ketamine	↑
OLA + KET	–	↑	–	↑	Antagonism to ketamine	–

The effect of the acute administration of 1MeTIQ (25 or 50 mg/kg i.p.) or olanzapine (OLA) on ketamine-induced (KET) changes in dopamine (DA) and noradrenaline (NA) release and levels of its metabolites in the rat striatum. ↑ increase, ↓ decrease, – lack change

## Discussion

In the present study, we used the NOR test to investigate the pro-cognitive potential of acute 1MeTIQ administration in a ketamine model of schizophrenia. Moreover, 1MeTIQ activity was compared to the results obtained by the administration of an atypical neuroleptic, olanzapine. In this paper, we demonstrated, for the first time, that the therapeutic activity of 1MeTIQ is comparable to that of olanzapine in the ketamine-induced impairment of memories in rats.

In the 1950s, the dopamine theory of schizophrenia was established based on excessive dopamine transmission (Delay et al. 1952; Carlsson and Lindqvist 1963). As it demonstrated by Pezze et al. (2007) both type of dopaminergic receptors (D1 and D2) are involved in cognitive processes. However, they modulate different aspects of performance. However, some researchers have suggested NA (Hornykiewicz 1986; Yamamoto et al. 1994; Maletic et al. 2017) as a contributor to the development of the illness because positive symptoms are aggravated by NA agonists and ameliorated by NA antagonists (Yamamoto and Hornykiewicz 2004). Based on pharmacological, biochemical and psychophysiological evidence, it has been proposed that both positive and negative symptoms may be the result of the dysregulation of the noradrenergic system. It was demonstrated that noradrenaline was elevated in both the blood plasma and cerebrospinal fluid of patients with schizophrenia, as well as noradrenergic markers being elevated in the postmortem in the brain (Kemali et al. 1982; Farley et al. 1978). Moreover, many studies indicate the essential participation of noradrenaline and its receptors (α1, α2, β) in the striatum, hippocampus and frontal cortex in cognitive and memory functions (Borodovitsyna et al. 2017). However, different noradrenergic receptors may play opposite roles in cognition; for example, the activation of the noradrenergic β receptor is necessary for both contextual and spatial memory consolidation and retrieval (Zhang et al. 2013), whereas the opposite effect was observed after the activation of the α1 receptor disrupted these functions (Hillman et al. 2009). It was demonstrated that the local application of the α1 receptor antagonist prazosine into the hippocampus (the dental gyrus) increased the rate of active avoidance learning; in contrast, this behaviour was acquired more slowly when phenylephrine, an α1 agonist, was administered (Zhan et al. 2016).

The NOR test has emerged as the most popular task for assessing rodent memory in terms of the ability to recognise a previously presented object (Ennanceur and Delacour 1988). The NOR test is based on rodents’ self-motivation to approach and explore new, non-threatening items using multiple senses, which is easily quantifiable. Healthy animals, during the test session, exhibit a preference for exploring the novel object significantly more than the familiar one (Cohen and Stackman Jr. 2015; Pezze et al. 2015). In accordance with previous studies (Verma and Moghaddam 1996; Adler et al. 1998; Kos et al. 2006), ketamine at sub-anaesthetic doses produced memory deficits. In our study, the administration of ketamine caused animals to spend less time exploring the novel object in the T2 phase of the NOR test (Fig. 1b; Fig. 3b), which indicates memory impairment. This result is consistent with those of other studies using the NOR test to evaluate cognitive deficits (Goulart et al. 2010; Nikiforuk et al. 2013; Pitsikas et al. 2008). Ketamine also decreases the preference index (which measures the ability of an animal to recognise the same object at different points in time) and the discrimination index (which quantifies the ability of an animal to discriminate between two different objects that are presented at the same time (D’Isa et al. 2014) (Fig. 2a, b; Fig. 4a, b). This result suggests the animals’ inability to recognise the familiar object in the T2 phase of the test.

In the present study, we demonstrated that 1MeTIQ exhibits pro-cognitive properties, as measured in the NOR test in a widely used rodent ketamine model of schizophrenia. This is the first study verifying the effect of 1MeTIQ on memory in rats and comparing its action to the effect of olanzapine, which is widely used in the treatment of schizophrenia. In the present study, we observed reduction of the total time of exploration of both objects after treatment with both doses of 1MeTIQ and olanzapine compare to control (saline group) in T1 phase (Fig. 1a; Fig. 3a). This effect is associated with a decrease in the locomotor activity of animals after administration of 1MeTIQ and olanzapine in T1 phase. Interestingly, we did not observe differences in the locomotor activity of rats in the T2 phase. (data not shown). In T1 phase we compare the times of exploration of object 1 and object 2 within one group to see if animals in particular treated group statistically differed in exploration times of two identical objects, to verify if non-specific preference for any object occurs.

We showed that 1MeTIQ, both at doses of 25 mg/kg and 50 mg/kg, reversed the effect of ketamine in T2 of the NOR test and increased the difference between the exploration times for the familiar and novel objects, with rats showing similar times to rats in the control group (Fig. 1b; Fig. 3b). In this phase, total exploration time of both objects (FO and NO) after treatment with both doses of 1MeTIQ and olanzapine was similar to the control group. This effect suggests successful recognition, which is demonstrated by rodents spending more time exploring the novel object than the familiar object during the T2 phase (Ennanceur and Delacour 1988). The effect of 1MeTIQ is comparable to the action of olanzapine, which reversed the ketamine effect in a congruent manner and improved performance in the NOR test. Clinical and pre-clinical studies indicated that olanzapine has been shown to improve cognitive functions, including memory (Wolff and Leander 2003; He et al. 2005; Mahmoud et al. 2019; Desamericq et al. 2014; Guo et al. 2011; Gurpegui et al. 2007; Meltzer and McGurk 1999; McGurk et al. 2004; Mutlu et al. 2011; Rajagopal et al. 2014; Babic et al. 2018); however, there are studies reporting that atypical antipsychotic drugs may cause cognitive impairment (Skarsfeldt 1996; Levin et al. 2005). 1MeTIQ administered at a dose of 25 mg/kg reversed the effect of ketamine and increased both preference and discrimination indexes (Fig. 2a, b), improving the animals’ performance in the NOR test. However, 1MeTIQ administered at a dose of 50 mg/kg tended to increase the DI and PI (compared to ketamine-treated animals), but this effect was not statistically significant. Surprisingly, olanzapine did not reverse the ketamine effect and did not increase the PI or DI significantly compared to the ketamine-treated group. However, there was a tendency to increase the index value in olanzapine-treated animals (Fig. 4a, b). In animal models of schizophrenia tested in the NOR task, olanzapine has been reported to restore the control value of PI (Mutlu et al. 2011) and increase the exploration time for novel objects (Snigdha et al. 2010), which seems to be consistent with our results from behavioural testing. To explain the NOR test performance, we carried out an in vivo microdialysis study and verified the release of monoamines in the rat striatum.

As shown by our present study, ketamine did not change dopamine release in the rat striatum (Fig. 5a; Fig. 7a), although it significantly decreased the concentration of its metabolites, DOPAC and HVA (Fig. 5c, d; Fig. 7c, d). However, the results obtained by other authors demonstrated that ketamine causes elevated glutamate and dopamine release in the prefrontal cortex and limbic regions, which leads to cognitive disorders (Moghaddam et al. 1997; Razoux et al. 2007). However, other authors have shown that an elevated dopamine concentration is beneficial for cognitive control (Colzato et al. 2014, 2016) and cognitive flexibility (Steenbergen et al. 2015). It is interesting that the beneficial effect of D1 receptor stimulation is found in the fronto-striatal network implicated in the control of 5CSRT performance, but not within the dorsolateral striatum (Pezze et al. 2007). Our results indicated that 1MeTIQ given alone and combined with ketamine significantly elevated dopamine release (approx. 200% and 250%, respectively) (Fig. 5a; Fig. 7a). In addition, equally important and characteristic of 1MeTIQ activity, 3-MT levels very sharply increased both in the 1MeTIQ-treated group (500%) and in the 1MeTIQ + ketamine combined group (up to 1000%). Such a powerful increase in 3-MT levels in the striatum may have essential therapeutic implications because this extraneuronal dopamine metabolite plays a physiological role in the brain as an inhibitory regulator of catecholaminergic activity. We have previously demonstrated that 3-MT binds to the α1 and striatal dopamine D1 and D2 receptors in the nanomolar concentration range (Antkiewicz-Michaluk et al. 2008). In contrast to 1MeTIQ, olanzapine increases the MAO-dependent oxidation pathway of dopamine metabolism and increases the DOPAC (approx. 300%) and HVA (approx. 250%) levels in the rat striatum (Fig. 9c, d). Olanzapine, as an atypical neuroleptic drug, acts as an antagonist of dopamine (D_2_, D_3_, D_4_), serotonin (5-HT_2A_, 5-HT_2C_, 5-HT_6_) and noradrenergic (α1) receptors (Mauri et al. 2014); thus, the receptor profile for olanzapine and 1MeTIQ seems to be similar considering the high affinity of 3-MT to dopamine and noradrenergic receptors (Antkiewicz-Michaluk et al. 2008).

There is evidence that both DA and NA play important roles in working memory. Clinical and animal studies have indicated that both excessive and reduced concentrations of DA and NA can induce cognitive impairment and deficits in working memory (Arnsten 1997; Inagaki et al. 2010; Murphy et al. 1996a, 1996b; Zahrt et al. 1997). These results suggest that DA has an inverted U-shaped dose-response relationship at D1 receptors (Vijayraghavan et al. 2007). NA increased working memory via alpha-2 adrenergic receptors, while the stimulation of alpha-1 receptors induced opposing effects (Arnsten 1997). Memory impairment appears during stress and after the administration of D1 and alpha-1 agonists, which lead to the release of DA and NA, respectively. The abovementioned data suggest that the optimal function of the frontal cortex (FCX) is dependent on appropriate levels of DA and NA (Arnsten 1997).

Elevated noradrenaline is thought to be a feature of schizophrenia, and it is possible that impaired frontal noradrenaline signalling contributes to cognitive deficits seen in the illness (Friedman et al. 1999; Fitzgerald 2014). Our in vivo microdialysis study indicated that ketamine produced a time-dependent increase in NA release in the rat striatum (up to 400%) (Fig. 6a; Fig. 8a). The probable mechanism responsible for this effect of ketamine is the inhibition of NMDA receptors located on GABAergic interneurons, which ultimately results in the disinhibition of other neurons, e.g., noradrenergic neurons (Boultadakis and Pitsikas 2011). There is evidence that NA modulates the efficacy of glutamatergic transmission by activating G protein coupled adrenergic receptors (Scheiderer et al. 2004).

In the present paper, we demonstrated that the behavioural and biochemical effects of ketamine were completely antagonised by 1MeTIQ (in both doses used) and by the atypical neuroleptic olanzapine. Next, the question arises: what could be the pro-cognitive mechanism of action for olanzapine and 1MeTIQ? It seems that the receptor profile for both investigated drugs, especially the antagonism to the noradrenergic α1 receptor, as mentioned above, may play an essential role in their pro-cognitive action. Moreover, 1MeTIQ and olanzapine can modulate the activity of GABA medium spiny neurons (MSNs) within the rat striatum; these neurons express dopamine D1 and D2 receptors and, consequently, antagonise the ketamine-induced release of NA (Ztaou and Amalric 2019**)**. It is also important to mention that 1MeTIQ, as a possible pro-cognitive drug, in contrast to olanzapine, expresses beneficial neuroprotective activities in the brain as an inhibitor of MAO-dependent dopamine oxidation and shifts dopamine catabolism towards COMT-dependent methylation, leading to the accumulation of 3-MT. As it was mention above, 3-MT an extraneuronal dopamine metabolite possesses the distinct physiological inhibitory effect on the catecholaminergic system activity within the brain (Antkiewicz-Michaluk et al. 2008; Wąsik et al. 2010).

## References

[CR1] Adler CM, Goldberg TE, Malhotra AK, Pickar D, Breier A (1998). Effects of ketamine on thought disorder, working memory, and semantic memory in healthy volunteers. Biol Psychiatry.

[CR2] Antkiewicz-Michaluk L, Michaluk J, Mokrosz M, Romanska I, Lorenc-Koci E, Ohta S, Vetulani J (2001). Different action on dopamine catabolic pathways of two endogenous 1,2,3,4-tetrahydroisoquinolines with similar antidopaminergic properties. J Neurochem.

[CR3] Antkiewicz-Michaluk L, Lazarewicz JW, Patsenka A, Kajta M, Zieminska E, Salinska E, Wasik A, Golembiowska K, Vetulani J (2006). The mechanism of 1,2,3,4-tetrahydroisoquinolines neuroprotection: the importance of free radicals scavenging properties and inhibition of glutamate-induced excitotoxicity. J Neurochem.

[CR4] Antkiewicz-Michaluk L, Filip M, Michaluk J, Romańska I, Przegaliński E, Vetulani J (2007). An endogenous neuroprotectant substance, 1-methyl-1,2,3,4-tetrahydroisoquinoline (1MeTIQ), prevents the behavioral and neurochemical effects of cocaine reinstatement in drug-dependent rats. J Neural Transm.

[CR5] Antkiewicz-Michaluk L, Ossowska K, Romańska I, Michaluk J, Vetulani J (2008). 3-Methoxytyramine, an extraneuronal dopamine metabolite play a physiological role in the brain as an inhibitory regulator of catecholaminergic activity. Eur J Pharmacol.

[CR6] Antkiewicz-Michaluk L, Wąsik A, Michaluk J (2014). 1-Methyl-1,2,3,4-tetrahydroisoquinoline, an endogenous amine with unexpected mechanism of action: new vistas of therapeutic application. Neurotox Res.

[CR7] Antunes M, Biala G (2012). The novel object recognition memory: neurobiology, test procedure, and its modifications. Cogn Process.

[CR8] Arnsten AF (1997). Catecholamine regulation of the prefrontal cortex. J Psychopharmacol.

[CR9] Aubry JM, Simon AE, Bertschy G (2000). Possible induction of mania and hypomania by olanzapine or risperidone: a critical review of reported cases. J Clin Psychiatry.

[CR10] Babic I, Gorak A, Engel M, Sellers D, Else P, Osborne AL, Pai N, Huang XF, Nealon J, Weston-Green K (2018). Liraglutide prevents metabolic side-effects and improves recognition and working memory during antipsychotic treatment in rats. J Psychopharmacol.

[CR11] Balu DT (2016). The NMDA receptor and schizophrenia: from pathophysiology to treatment. Adv Pharmacol.

[CR12] Bondi C, Matthews M, Moghaddam B (2012). Glutamatergic animal models of schizophrenia. Curr Pharm Des.

[CR13] Borodovitsyna Olga, Flamini Matthew, Chandler Daniel (2017). Noradrenergic Modulation of Cognition in Health and Disease. Neural Plasticity.

[CR14] Boultadakis A, Pitsikas N (2011). Anesthetic ketamine impairs rats’ recall of previous information: the nitric oxide synthase inhibitor N-nitro-L-arginine methylester antagonizes this ketamine-induced recognition memory deficits. Anesthesiology.

[CR15] Brandao-Teles C, Martins-de-Souza D, Guest PC, Cassoli JS (2017). MK-801-treated oligodendrocytes as a cellular model to study schizophrenia. Adv Exp Med Biol.

[CR16] Bubeniková-Valesová V, Horácek J, Vrajová M, Höschl C (2008). Models of schizophrenia in humans and animals based on inhibition of NMDA receptors. Neurosci Biobehav Rev.

[CR17] Cadinu D, Grayson B, Podda G (2018). NMDA receptor antagonist rodent models for cognition in schizophrenia and identification of novel drug treatments, an update. Neuropharmacology.

[CR18] Carlsson A, Lindqvist M (1963). Effect of chlorpromazine or haloperidol on formation of 3-methoxytyramine and normetanephrine in mouse brain. Acta Pharmacol Toxicol (Copenh).

[CR19] Chiueh CC, Miyake H, Peng MT (1993). Role of dopamine autoxidation, hydroxyl radical generation, and calcium overload in underlying mechanisms involved in MPTP-induced parkinsonism. Adv Neurol.

[CR20] Cohen SJ, Stackman RW (2015). Assesing rodent hippocampal involvement in the novel object recognition task. Behav Brain Res.

[CR21] Colzato LS, van den Wildenberg WP, Hommel B (2014). Cognitive control and the COMT Val^158^Met polymorphism: genetic modulation of videogame training and transfer to task-switchng efficiency. Psychol Res.

[CR22] Colzato LS, Steenbergen L, Sellaro R (2016). Effetcs of l-tyrosine on working memory and inhibitory control are determined by DRD2 genotypes: a randomized cotrnolled trial. Cortex.

[CR23] Coyle JT (2012). NMDA receptor and schizophrenia: a brief history. Schizophr Bull.

[CR24] D’Isa R, Brambilla R, Fasano S (2014). Behavioral methods for the study of the Ras-ERK pathway in memory formation and consolidation: passive avoidance and novel object recognition tests. Methods Mol Biol.

[CR25] Danion JM, Rizzo L, Bruant A (1999). Functional mechanisms underlying impaired recognition memory and conscious awareness in patient with schizophrenia. Arch Gen Psychiatry.

[CR26] Delay J, Deniker P, Harl JM (1952). Therapeutic use in psychiatry of phenothiazine of central elective action (4560 RP). Ann Med Psychol (Paris).

[CR27] Desamericq G, Schurhoff F, Meary A (2014). Long-term neurocognitive effects of antipsychotics in schizophrenia: a network meta-analysis. Eur J Clin Pharmacol.

[CR28] Duncan GE, Sheitman BB, Lieberman JA (1999). An integrated view of pathophysiological models of schizophrenia. Brain Res Brain Res Rev.

[CR29] Ennaceur A (2010). One-trial object recognition in rats and mice: methodological and theoretical issues. Behav Brain Res.

[CR30] Ennanceur A, Delacour J (1988). A new one-trial test for neurobiological studies of memory in rats. I. Behavioral data. Behav Brain Res.

[CR31] Faraone SV, Seidman LJ, Kremen WS, Toomey R, Pepple JR, Tsuang MT (2000). Neuropsychologic functioning among the nonpsychotic relatives of schizophrenic patients: the effect of genetic loading. Biol Psychiatry.

[CR32] Farley IJ, Price KS, McCullough E (1978). Norepinephrine in chronic paranoid schizophrenia: above-normal levels in limbic forebrain. Science.

[CR33] Fitzgerald PJ (2014). Is elevated norepinephrine an etiological factor in some cases of schizophrenia?. Psychiatry Res.

[CR34] Friedman JI, Adler DN, Davis KL (1999). The role of norepinephrine in the pathophysiology of cognitive disorders: potential applications to the treatment of cognitive dysfunction in schizophrenia and Alzheimer’s disease. Biol Psychiatry.

[CR35] Gilmour G, Dix S, Fellini L, Gastambide F, Plath N, Steckler T, Talpos J, Tricklebank M (2012). NMDA receptors, cognition and schizophrenia – testing the validity of the NMDA receptor hypofunction hypothesis. Neuropharmacology.

[CR36] Ginos JZ, Doroski D (1979). Dopaminergic antagonists: effects of 1,2,3,4-tetrahydroisoquinoline and its N-methyl and N-propyl homologs on apomorphine- and L-dopa-induced behavioral effects in rodents. J Pharmacol Exp Ther.

[CR37] Goulart BK, de Lima MN, de Farias CB (2010). Ketamine impairs recognition memory consolidation and prevents learning-induced increase in hippocampal brain-derived neurotrophic factor levels. Neuroscience.

[CR38] Guo X, Zhai J, Wei Q, Twamley EW, Jin H, Fang M, Hu M, Zhao J, Early-stage Schizophrenia Outcome Study (ESOS) Investigators (2011). Neurocognitive effects of first- and second-generation antipsychotic drugs in early-stage schizophrenia: a naturalistic 12-month follow-up study. Neurosci Lett.

[CR39] Gurpegui M, Alvarez E, Bousono M (2007). Effect pf olanzapine or risperidone treatment on some cognitive functions in one-year follow-up of schizophrenia outpatients with prominent negative symptoms. Eur Neuropsychopharmacol.

[CR40] He J, Yang Y, Xu H, Zhang X, Li XM (2005). Olanzapine attenuates the okadaic acid-induced spatial memory impairment and hippocampal cell death in rats. Neuropsychopharmacology.

[CR41] Heckers S, Curran T, Goff D, Rauch SL, Fischman AJ, Alpert NM, Schacter DL (2000). Abnormalities in the thalamus and prefrontal cortex during episodic object recognition in schizophrenia. Biol Psychiatry.

[CR42] Heinrichs RW, Zakzanis KK (1998). Neurocognitive deficit in schizophrenia: a quantitive review of the evidence. Neuropsychology.

[CR43] Hillman KL, Lei S, Doze VA, Porter JE (2009). Alpha-1A adrenergic receptor activation increases inhibitory tone in CA1 hippocampus. Epilepsy Res.

[CR44] Hornykiewicz O (1986). Brain noradrenaline and schizophrenia. Prog Brain Res.

[CR45] Inagaki T, Gautreaux C, Luine V (2010). Acute estrogen treatment facilitates recognition memory consolidation and alters monoamine levels in memory-related brain areas. Horm Behav.

[CR46] Kemali D, Del Vecchio M, Maj M (1982). Increased noradrenaline levels in CSF and plasma of schizophrenic patients. Biol Psychiatry.

[CR47] Kos T, Popik P, Pietraszek M, Schäfer D, Danysz W, Dravolina O, Blokhina E, Galankin T, Bespalov AY (2006). Effect of 5-HT3 receptor antagonists MDL 72222 on behaviors induced by ketamine in rats and mice. Eur Neuropsychopharmacol.

[CR48] Krabbendam L, Marcelis M, Delespaul P, Jolles J, van Os J (2001). Single or multiple familial cognitive risk factors in schizophrenia?. Am J Med Genet.

[CR49] Krystal JH, D’Souza DC, Petrakis IL (1999). NMDA agonists and antagonists as probes of glutamatergic dysfunction and pharmacotherapies in neuropsychiatric disorders. Harv Rev Psychiatry.

[CR50] Kuszczyk MA, Sadowski MJ, Antkiewicz-Michaluk L, Lazarewicz JW (2013). 1MeTIQ provides protection against Aβ-induced reduction of surface expression of synaptic proteins and inhibits H2O2-induced oxidative stress in primary hippocampal neurons. Neurotox Res.

[CR51] Lesh TA, Niendam TA, Minzenberg MJ, Carter CS (2011). Cognitive control deficits in schizophrenia: mechanisms and meaning. Neuropsychopharmacology.

[CR52] Levin ED, Petro A, Beatty A (2005). Olanzapine interactions with nicotine and mecamylamine in rats: effects on memory function. Neurotoxicol Teratol.

[CR53] Mahmoud GS, Sayed SA, Abdelmawla SN, Amer MA (2019). Positive effects of systemic sodium benzoate and olanzapine treatment on activities of daily life, spatial learning and working memoy in ketamine-induced rat model of schizophrenia. Int J Physiol Pathophysiol Pharmacol.

[CR54] Maletic V, Eramo A, Gwin K, Offord SJ, Duffy RA (2017). The role of norepinephrine and its α-adrenergic receptors in the pathophysiology and treatment of major depressive disorder and schizophrenia: a systematic review. Front Psychiatry.

[CR55] Mathiasen JR, DiCamillo A (2010) Novel object recognition in the rat: a facile assay for cognitive function. Curr Protoc Pharmacol chapter 5, unit 5.59. 10.1002/0471141755.ph0559s49 10.1002/0471141755.ph0559s49 22294372

[CR56] Mauri MC, Paletta S, Maffini M, Colasanti A, Dragogna F, di Pace C, Altamura AC (2014). Clinical pharmacology of atypical antipsychotics: an update. EXCLI J.

[CR57] McGurk SR, Lee MA, Jayathilake K, Meltzer HY (2004). Cognitive effects of olanzapine treatment in schizophrenia. MedGenMed.

[CR58] Meltzer HY, McGurk SR (1999). The effects of clozapine, risperidone and olanzapine on cognitive function in schizophrenia. Schizophr Bull.

[CR59] Miller JW, Selhub J, Joseph JA (1996). Oxidative damage caused by free radicals produced during catecholamine autoxidation: protective effects of O-methylation and melatonin. Free Radic Biol Med.

[CR60] Moghaddam B, Adams B, Verma A, Daly D (1997). Activation of glutamatergic neurotransmission by ketamine: a novel step in the pathway from NMDA receptor blockade to dopaminergic and cognitive disruptions associated with the prefrontal cortex. J Neurosci.

[CR61] Możdżeń E, Wąsik A, Romańska I, Michaluk J, Antkiewicz-Michaluk L (2017). Antidepressant-like effect of 1,2,3,4-tetrahydroisoquinoline and its methyl derivative in animals models of depression. Pharmacol Rep.

[CR62] Murphy BL, Arnsten AF, Goldman-Rakic PS, Roth RH (1996). Ncreased dopamine turnover in the prefrontal cortex impairs spatial working memory performance in rats and monkeys. Proc Natl Acad Sci U S A.

[CR63] Murphy BL, Arnsten AF, Jentsch JD, Roth RH (1996). Dopamine and spatial working memory in rats and monkeys: pharmacological reversal of stress-induced impairment. J Neurosci.

[CR64] Mutlu O, Ulak G, Celikyurt IK (2011). Effects of olanzapine, sertindole and clozapine on MK-801 induced visual memory deficits in mice. Pharmacol Biochem Behav.

[CR65] Nakazawa K, Jeevakumar V, Nakao K (2017). Spatial and temporal boundaries of NMDA receptor hypofunction leading to schizophrenia. NPJ Schizophr.

[CR66] Neill JC, Barnes S, Cook S, Grayson B, Idris NF, McLean S, Snigdha S, Rajagopal L, Harte MK (2010). Animal models of cognitive dysfunction and negative symptoms of schizophrenia: focus on NMDA receptor antagonism. Pharmacol Ther.

[CR67] Nikiforuk A, Fijał K, Potasiewicz A, Popik P, Kos T (2013). The 5-hydroxytryptamine (serotonin) receptor 6 agonist EMD 386088 ameliorates ketamine-induced deficits in attentional set shifting and novel object recognition, but not in the prepulse inhibition in rats. J Psychopharmacol.

[CR68] Nikiforuk A, Potasiewicz A, Kos T, Popik P (2016). The combination of memantine and galantamine improves cognition in rats: the synergistic role of the α7 nicotinic acetylcholine and NMDA receptors. Behav Brain Res.

[CR69] Patsenka A, Antkiewicz-Michaluk L (2004). Inhibition of rodent brain monoamine oxidase and tyrosine hydroxylase by endogenous compounds – 1,2,3,4-tetrahydroisoquinoline alkaloids. Pol J Pharmacol.

[CR70] Pezze MA, Dalley JW, Robbins TW (2007). Differential roles of dopamine D1 and D2 receptors in the nucleus accumbens in attentional performance on the five-choice serial reaction time task. Neuropsychopharmacology.

[CR71] Pezze MA, Marshall HJ, Fone KC, Cassaday HJ (2015). Dopamine D1 receptor stimulation modulates the formation and retrieval of novel object recognition memory: role of the prelimbic cortex. Eur Neuropsychopharmacol.

[CR72] Pietraszek M, Michaluk J, Romańska I, Wasik A, Gołembiowska K, Antkiewicz-Michaluk L (2009). 1-Methyl-1,2,3,4-tetrahydroisoquinoline antagonizes a rise in brain dopamine metabolism, glutamate release in frontal cortex and locomotor hyperactivity produced by MK-801 but not the disruptions of prepulse inhibition, and impairment of working memory in rat. Neurotox Res.

[CR73] Pitsikas N, Boultadakis A, Sakellaridis N (2008). Effects of sub-anesthetic doses of ketamine on rat’s spatial and non-spatial recognition memory. Neuroscience.

[CR74] Purdon S, Jones B, Stip E, Labelle A, Addington D, David SR, Breier A, Tollefson GD (2000). Neuropsychological change in early phase schizophrenia during 12 months of treatment with olanzapine, risperidone, or haloperidol. Arch Gen Psychiatry.

[CR75] Rajagopal L, Massey BW, Huang M, Oyamada Y, Meltzer HY (2014). The novel object recognition test in rodents in relation to cognitive impairment in schizophrenia. Curr Pharm Des.

[CR76] Razoux F, Garcia R, Léna I (2007). Ketamine, at a dose that disrupts motor behavior and latent inhibition, enhances prefrontal cortex synaptic efficacy and glutamate release in the nucleus accumbens. Neuropsychopharmacology.

[CR77] Reger ML, Hovda DA, Giza CC (2009). Ontogeny of rat recognition memory measured by the novel object recognition task. Dev Psychobiol.

[CR78] Riedel WJ, Blokland A, Kantak K, Wettstein J (2015). Declarative memory. Cognitive enhancement. Handbook of Experimental Pharmacology.

[CR79] Rogóż Z, Skuza G (2011). Anxiolytic-like effects of olanzapine, risperidone and fluoxetine in the elevated plus-maze test in rats. Pharmacol Rep.

[CR80] Scheiderer CL, Dobrunz LE, McMahon LL (2004). Novel form of long-term synaptic depression in rat hippocampus induced by actiovation of α1 adrenergic receptors. J Neurophysiol.

[CR81] Silvers JM, Harrod SB, Mctutus CF, Booze RM (2007). Automation of the novel object recognition task for use in adolescent rats. J Neurosci Methods.

[CR82] Sivakumaran MH, Mackenzie AK, Callan IR et al (2018) The discrimination ratio derived from novel object recognition task as a measure of recognition memory sensitivity, not bias. Sci Rep 8. 10.1038/s41598-018-30030-7 10.1038/s41598-018-30030-7 PMC6070491 30069031

[CR83] Skarsfeldt T (1996). Differential effect of antipsychotics on place navigation of rats in the Morris water maze. A comparative study between novel and reference antipsychotics. Psychopharmacology.

[CR84] Snigdha S, Horiguchi M, Huang M (2010). Attenuation of phencyclidine-induced object recognition deficits by the combination of atypical antipsychotic drugs and pimavanserin (ACP 103), a 5-hydroxytryptamine(2A) receptor inverse agonist. J Pharmacol Exp Ther.

[CR85] Steenbergen L, Sellato R, Hommel B, Colzato LS (2015). Tyrosine promotes cognitive flexibility: evidence from proactive vs. reactive control during task switching performance. Neuropsychologia.

[CR86] Toulopoulou T, Rabe-Hesketh S, King H, Murray RM, Morris RG (2003). Episodic memory in schizophrenic patients and their relatives. Schizophr Res.

[CR87] Verma A, Moghaddam B (1996). NMDA receptor antagonists impair prefrontal cortex function as asesed via spatial delayed alternation performance in rats: modulation by dopamine. J Neurosci.

[CR88] Vetulani J, Nalepa I, Antkiewicz-Michaluk L, Sansone M (2001). Opposite effect of simple tetrahydroisoquinolines on amphetamine- and morphine-stimulated locomotor activity in mice. J Neural Transm (Vienna).

[CR89] Vetulani J, Antkiewicz-Michaluk L, Nalepa I, Sansone M (2003). A possible physiological role for cerebral tetrahydroisoquinolines. Neurotox Res.

[CR90] Vijayraghavan S, Wang M, Birnbaum SG, Williams GV, Arnsten AF (2007). Inverted-U dopamine D1 receptor actions on prefrontal neurons engaged in working memory. Nat Neurosci.

[CR91] Wąsik A, Antkiewicz-Michaluk L (2017). The mechanism of neuroprotective action of netural compounds. Pharmacol Rep.

[CR92] Wąsik A, Romańska I, Antkiewicz-Michaluk L (2010). Important role of 3-methoxytyramine in the inhibition of cocaine sensitization by 1-methyl-1,2,3,4-tetrahydroisoquinoline: an in vivo microdialysis study. Pharmacol Rep.

[CR93] Wąsik A, Romańska I, Antkiewicz-Michaluk L (2016). Comparison of the effects of acute and chronic administration of tetrahydroisoquinoline amines on the in vivo dopamine release: a microdialysis study in the rat striatum. Neurotox Res.

[CR94] Wąsik A, Białoń M, Żarnowska M, Antkiewicz-Michaluk L (2019). Comparison of the effects of 1MeTIQ and olanzapine on performance in the elevated plus maze test and monoamine metabolism in the brain after ketamine treatment. Pharmacol Biochem Behav.

[CR95] Weickert CS, Fung SJ, Catts VS (2013). Molecular evidence of N-methyl-D-aspartate receptor hypofunction in schizophrenia. Mol Psychiatry.

[CR96] Wolff MC, Leander JD (2003). Comparison of the effects of antipsychotic on delayed radial maze task in the rat. Psychopharmacology.

[CR97] Yamakawa T, Kotake Y, Fujitani M (1999). Regional distribution of parkinsonism-preventing endogenous tetrahydroisoquinoline derivatives and an endogenous parkinsonism-preventing substance-synthesizing enzyme in monkey brain. Neurosci Lett.

[CR98] Yamamoto K, Hornykiewicz O (2004). Proposal for a noradrenaline hypothesis of schizophrenia. Prog Neuro-Psychopharmacol Biol Psychiatry.

[CR99] Yamamoto K, Ozawa N, Shinba T, Hoshino T, Yoshii M (1994). Possible noradrenergic dysfunction in schizophrenia. Brain Res Bull.

[CR100] Yang AC, Tsai SJ (2017). New targets for schizophrenia treatment beyond the dopamine hypothesis. Int J Mol Sci.

[CR101] Zahrt J, Taylor JR, Mathew RG, Arnsten AF (1997). Supranormal stimulation of D1 receptors in the rodent prefrontal cortex impairs spatial working memory performance. J Neurosci.

[CR102] Zhan JLSY, Wang GXLD, Li YS, Jin QH (2016). Alpha 1-adrenoceptors in the hippocampal dentate gyrus involved in learning-dependent long-term potentiation during active-avoidance learning in rats. Neuroreport.

[CR103] Zhang L, Ouyang M, Ganellin CR, Thomas SA (2013). The slow afterhyperpolarization: a target of β1-adrenergic signaling in hippocampus-dependent memory retrieval. J Neurosci.

[CR104] Ztaou S, Amalric M (2019). Contribution of cholinergic interneurons to striatal pathophysiology in Parkinson’s disease. Neurochem Int.

